# Identifying novel associations in GWAS by hierarchical Bayesian latent variable detection of differentially misclassified phenotypes

**DOI:** 10.1186/s12859-020-3387-z

**Published:** 2020-05-07

**Authors:** Afrah Shafquat, Ronald G. Crystal, Jason G. Mezey

**Affiliations:** 1 Department of Computational Biology, Cornell University, Ithaca, NY USA; 2 Department of Genetic Medicine, Weill Cornell Medicine, New York, NY USA; 3 Department of Medicine, Weill Cornell Medicine, New York, NY USA

**Keywords:** GWAS, Misclassification, Bayesian, Hierarchical latent variable models, MCMC, UK Biobank

## Abstract

**Background:**

Heterogeneity in the definition and measurement of complex diseases in Genome-Wide Association Studies (GWAS) may lead to misdiagnoses and misclassification errors that can significantly impact discovery of disease loci. While well appreciated, almost all analyses of GWAS data consider reported disease phenotype values as is without accounting for potential misclassification.

**Results:**

Here, we introduce *Phenotype Latent variable Extraction of disease misdiagnosis* (PheLEx), a GWAS analysis framework that learns and corrects misclassified phenotypes using structured genotype associations within a dataset. PheLEx consists of a hierarchical Bayesian latent variable model, where inference of differential misclassification is accomplished using filtered genotypes while implementing a full mixed model to account for population structure and genetic relatedness in study populations. Through simulations, we show that the PheLEx framework dramatically improves recovery of the correct disease state when considering realistic allele effect sizes compared to existing methodologies designed for Bayesian recovery of disease phenotypes. We also demonstrate the potential of PheLEx for extracting new potential loci from existing GWAS data by analyzing bipolar disorder and epilepsy phenotypes available from the UK Biobank. From the PheLEx analysis of these data, we identified new candidate disease loci not previously reported for these datasets that have value for supplemental hypothesis generation.

**Conclusion:**

PheLEx shows promise in reanalyzing GWAS datasets to provide supplemental candidate loci that are ignored by traditional GWAS analysis methodologies.

## Background

Identification of statistical associations between phenotypes and genotypes within Genome-wide Association Studies (GWAS) has resulted in the detection of numerous candidate genetic loci that may impact human diseases and other aspects of human physiology [1, 2]. Since the first major GWAS were published [3–6], there has been an increased realization that for many diseases and traits, it will be challenging to identify the bulk of contributing genetic loci due to the nature of genetic effects where issues include small allelic effect sizes, genetic and environmental interactions, and segregation of contributing loci for rare alleles [7, 8]. This realization has driven improved strategies for GWAS discovery including consortium studies with large sample sizes that can detect small effect size loci [9–11], sampling of understudied populations to identify loci with differential genetic and environmental impacts [12–15], and whole-genome sequencing of individuals to assess the impact of rare alleles [16–21]. These GWAS design strategies have been paralleled and complemented by continued innovation in GWAS analysis methodologies, including methods that detect epistatic interactions among genetic loci [22–24] and genotype by environment interactions [25–28], as well as methods aimed to extract impact of loci with rare variants [29–33]. Together, these innovations in GWAS design and methodology have led to discovery of candidate loci where impact is particularly noticeable in diseases such as type 2 diabetes and schizophrenia where large-scale consortium studies have enabled isolation of numerous causal loci with low frequency and small effects [2, 34–37]. While these successes justify continuous investment in GWAS, it is clear that sustained rate of discovery of new loci for well-studied diseases and phenotypes will depend on innovative strategies that leverage underutilized aspects of GWAS.

A core aspect of GWAS that could be targeted with improved strategies is the phenotype, where there are opportunities for improved phenotype definition [38–40], measurement [41–43], and analysis [44–46]. It is well appreciated that the combination of inconsistency in methods used to diagnose disease [47, 48] and the application of imprecise measurement methodology [43] can introduce phenotyping errors that can reduce discovery potential of a GWAS [49–56]. For example, high misdiagnosis rates have been estimated for disease phenotypes such as Alzheimer’s disease and bipolar disorder which may be misdiagnosed with other forms of dementia and unipolar depression/borderline personality disorder, respectively, due to overlap of symptoms and/or lack of application of Diagnostic Systems Manual criteria [57–64]. As another example, patients with migraine, fibromyalgia, and psychogenic disorder may frequently be misdiagnosed with multiple sclerosis due to overlap of symptoms and mistakes in application of clinical and radiographic diagnostic criteria [65]. Though various strategies have been proposed to help address these issues through the processing of GWAS phenotype data [46, 66–69], a complementary strategy would be to consider alternative phenotypes derived from leveraging structure of total GWAS data. An underexplored analysis strategy that follows this approach is to consider misclassification of disease phenotypes [70–73], where error in disease phenotype would result in disease cases recorded as controls and vice versa [74]. Considering disease misdiagnosis rates [75, 76], there is significant potential for disease misclassification in GWAS phenotype data where even small numbers of these errors can have significant impact on GWAS statistical power and Type I errors [49, 50]. Methods that could reliably identify cases of misclassification in GWAS could be a promising approach for improving candidate loci discovery in GWAS, particularly when considering the potential for immediate impact and implementation at minimal cost.

There has been surprisingly little attention paid to phenotype misclassification analysis in GWAS, where misclassification errors could be inferred and corrected by making use of genotype associations with phenotype [49, 73, 77]. The only major published methods for GWAS analysis are Bayesian approaches for recovering non-differential misclassification (i.e. misclassification rates are considered the same for cases and non-cases/controls) [49, 73] and differential misclassification (misclassification rates are considered different for cases versus non-cases/controls) [77]. These methods and their extensions for gene expression data have since been applied in several studies to demonstrate potential benefits of misclassification analysis. Examples include identifying misdiagnosis of Alzheimer’s patients based on differential gene expression [78, 79], predicting disease subtypes in breast cancer using gene expression data [80], and finding misclassified individuals and estimating single nucleotide polymorphism (SNP) effects in simulated GWAS data [49, 73, 77]. Still, a number of gaps remain when considering these methods for the analysis of GWAS data. For example, only one misclassification method has been proposed for the analysis of GWAS data [49, 73, 77], where this method fails to account for inherent genetic relatedness and population structure in sampled GWAS populations. Given that ignoring this fundamental issue in GWAS analysis dramatically increases false positive rates, this seems a considerable omission [81, 82]. What’s more, this method was only shown to perform well on GWAS datasets simulated with an artificially high number of disease-associated SNPs out of the total number of SNPs (i.e. 150/1000) with genotype-specific disease-odds ratio in the range 4–10 [49, 77]. Such simulation scenarios provide an unrealistic picture of the algorithm’s expected performance on real GWAS datasets.

**Fig. 1:**
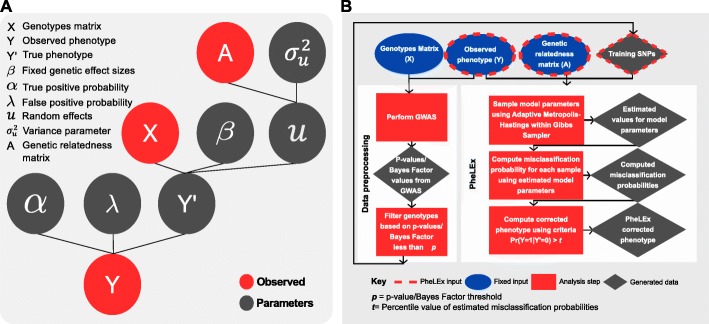
Overview of the PheLEx framework. **a** Underlying graphical model for the PheLEx method shows input: genotypes matrix *X,* observed phenotype *Y*, genetic relatedness matrix *A*, and architecture of model parameters used to infer misclassification probabilities in observed phenotype. **b** Overview of steps used to extract misclassification probabilities and produce corrected phenotypes for reanalysis using GWAS with the method PheLEx implemented in R package “phelex”. For detailed explanation, please refer to the main text

Here, we present a complete framework for Bayesian latent variable misclassification analysis that can be used to explore GWAS for new discoveries: Phenotype Latent variable Extraction of disease misdiagnosis (PheLEx) (Fig. 1). The core of PheLEx is a single modeling framework allowing for differential misclassification in GWAS phenotypes with an underlying full mixed model to account for genetic relatedness and population structure. When concentrating only on the problem of phenotype misclassification, we show that the PheLEx framework dramatically improved performance when analyzing simulated GWAS data that included realistic effect sizes and proportions of disease-associated genotypes in a genome-wide scan consistent with empirical observation [83–87]. Other applications of PheLEx include exploring differential patterns between misclassified and non-misclassified cases within GWAS datasets that may point to potential causes such as misdiagnosis or disease subtypes. We also propose a novel strategy for applying the PheLEx framework to explore new loci within a GWAS dataset by making use of misclassification probabilities for phenotype and strategic filtering of SNPs to improve accuracy and avoid model overfitting. We demonstrate the potential of this application by using PheLEx to analyze datasets for bipolar disorder and epilepsy phenotypes, where we discover “PheLEx” supplementary candidate loci that were not identified in the traditional analysis of these datasets and may contain information about disease-genotype associations. While caution and careful interpretation of such PheLEx driven discoveries is critical, these results demonstrate the potential of PheLEx for reanalyzing existing GWAS data to identify novel discoveries that may be explored for biological connections to disease phenotypes.

## Results

### PheLEx compared to existing methods

At present, there is only one existing misclassification framework designed for the analysis of GWAS data [49, 73, 77], referred to here as the “Rekaya” method or framework (or just as “Rekaya”). The Rekaya framework uses a Gibbs sampler to estimate misclassification rates (false positive rate and false negative rate) and identify misclassified samples from GWAS datasets, without accounting for random effects due to genetic relatedness/population structure. PheLEx addresses limitations of Rekaya [49, 73, 77] for disease misclassification by introducing: (i) filtration of potentially uninformative genotypes to address issue of the disproportionate (low) ratio of disease-associated SNPs in human GWAS, (ii) a more efficient Markov Chain Monte Carlo (MCMC) sampling algorithm, and (iii) accounting for genetic relatedness and population structure. Using Adaptive Metropolis-Hastings within Gibbs sampling allows PheLEx more flexibility in sampling from posterior probabilities. As accuracy of misclassification probability under the misclassification model depends on estimated function of SNP effects and typically most SNPs in a linkage disequilibrium (LD-) pruned GWAS dataset are not associated with the phenotype of interest, PheLEx filters out potentially uninformative SNPs by taking a subset of statistically significant GWAS genotypes as input, which provides significant advantages in terms of computational expense and accuracy in identifying misclassified samples. As genetic relatedness and population structure are a reality of most GWAS datasets [81, 82, 88], PheLEx accounts for these effects, which is critical for estimating accurate misclassification probabilities.

Beyond these methodology improvements for identifying misclassified phenotypes within a GWAS dataset, we also introduce a novel application of PheLEx for identifying new potential GWAS associations when making use of corrected phenotypes. PheLEx presents functions that can be used to estimate misclassification probabilities to produce a corrected phenotype, which in turn can be used to perform association analysis with the genotypes data. The corrected phenotype provides an alternative phenotype for association analysis, potentially allowing for new GWAS discoveries to be made with the new phenotype. Given that PheLEx uses a subset of genotypes to estimate misclassification probabilities, any SNPs not included in this training set (and not in LD with training SNPs) that are found to be statistically significant are considered novel PheLEx discoveries when analyzing the corrected phenotype. While clearly the value of such PheLEx discoveries depends on the correctness of the identified misclassifications and should therefore be considered in a separate class from the associations discovered when analyzing the original GWAS phenotype, PheLEx discoveries represent a supplemental set of hypotheses that can provide insight into genetic and biological connections to disease phenotypes.

### Performance impact of PheLEx components

To investigate benefit of filtering SNPs in the PheLEx framework compared to the Rekaya framework, we applied Rekaya to datasets simulated without genetic relatedness/population structure and used two different strategies for deciding on SNP inputs: (i) “Rekaya with PheLEx input” (filtered SNPs using PheLEx’s *p* threshold criteria) and (ii) “Rekaya” with unfiltered input where top 1000 SNPs with lowest *p*-values were used as input. As existing implementations of Rekaya suggest using all SNPs as input for the algorithm, the latter approach provides a way to understand the effect of adding potentially uninformative SNPs as training input. Performance was evaluated based on precision and recall metrics for identifying misclassified samples from simulated datasets. Comparison of Precision-Recall (PR) curves for “Rekaya with PheLEx input” and Rekaya (with unfiltered input) for these simulated data indicate that the PheLEx approach to filtering SNPs results in better performance overall (see Additional file 1: Figure S1 and Text S1). While Rekaya (with unfiltered input) performed slightly better than Rekaya with PheLEx input at lower misclassification levels, at higher levels of misclassification Rekaya with PheLEx input performed considerably better.

For these same simulated datasets, we also applied PheLEx-mm, a variant of PheLEx without the mixed model component (as in Rekaya), to isolate the impact on performance by incorporating the Adaptive Metropolis-Hastings step in MCMC algorithm of PheLEx compared to the full Gibbs sampler MCMC algorithm of Rekaya (see Additional file 1: Figure S1 and Text S1). When assessed by PR curves, PheLEx-mm had dramatically improved performance in identifying misclassified samples compared to “Rekaya with PheLEx input” and Rekaya, indicating this component of the PheLEx MCMC leads to a better exploration of the posterior of the underlying hierarchical model used in both methods and results in better performance overall.

**Fig. 2:**
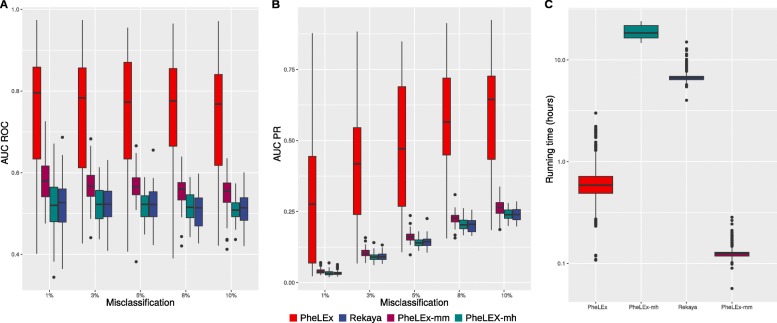
Performance comparison between PheLEx and other misclassification extraction methods in identifying misclassified samples in simulations. Misclassification extraction methods: PheLEx (red), PheLEx-mm (purple), PheLEx-mh (teal), and Rekaya (blue). **a** Box plots showing area under Receiver Operating Characteristic (ROC) curves (AUC ROC) (y-axis) for identifying misclassified samples across simulations against increasing misclassification rates (x-axis) for misclassification extraction methods. **b** Box plots showing area under Precision-Recall (PR) curves (AUC PR) (y-axis) for identifying misclassified samples across simulations against increasing misclassification rates (x-axis) for misclassification extraction methods. **c** Box plots for running time (hours) (y-axis) is shown for misclassification extraction methods (x-axis) across simulations

Finally, to provide a comparison of performance when considering GWAS data simulated with genetic relatedness/population structure, we compared four methods, PheLEx, PheLEx-mm, PheLEx-mh (a variant of PheLEx that includes the mixed model component but with an MCMC that does not include the Adaptive Metropolis-Hastings step), and Rekaya (Fig. 2 and Additional file 1: Text S2). The comparison of PheLEx versus PheLEx-mm and Rekaya assessed by Receiver Operating Characteristic (ROC) and PR curves indicates that not surprisingly accounting for population structure, when present in a GWAS, results in increased performance. The comparison of PheLEx and PheLEx-mh also confirms the observation that the Adaptive Metropolis-Hastings step is contributing to improved performance, where dropping this step when population structure is present results in performance similar to Rekaya.

### Identification of misclassified samples

We further investigated the overall performance improvement of the complete PheLEx framework compared to Rekaya by analyzing simulated datasets with genetic relatedness/population structure across varying degrees of misclassification (%false positives: 1, 3, 5, 8, and 10%). For these analyses, PheLEx outperformed Rekaya showing superior performance in both ROC curves and PR curves in identifying misclassified samples from misclassified phenotypes (Fig. 2). Area under curve (AUC) for ROC (AUC ROC) values computed across simulations were consistent on performance difference observed with PheLEx having the highest median AUC across misclassification levels, where median AUC values for PheLEx were higher than median AUC values for Rekaya (Table 1). Area under PR curve (AUC PR) values mirrored these results (Table 1).

**Table 1:** Performance evaluation of methods in identifying misclassified samples

Misclassification	Median AUC ROC^a^		Median AUC PR^b^	
	PheLEx	Rekaya	PheLEx	Rekaya
1%	0.795	0.527	0.276	0.0323
3%	0.783	0.523	0.418	0.09
5%	0.773	0.522	0.471	0.144
8%	0.776	0.514	0.565	0.205
10%	0.768	0.514	0.645	0.240

^a^AUC ROC = Area under Receiver Operating Characteristic curve

^b^AUC PR = Area under Precision Recall curve

Across increasing misclassification rates, the AUC ROC values were stable across increasing error in phenotype for both methods even though the number of training SNPs (that passed the *p*-value cut-off for filtering training SNPs) decreased with increasing misclassification rates (Additional file 1: Figure S2). AUC PR values increased for both methods across increasing misclassification rates. Overall, PheLEx consistently showed improved performance over Rekaya by use of Adaptive Metropolis-Hastings within Gibbs sampling algorithm and accounting for genetic relatedness/population structure instead of a full Gibbs sampler as used in existing methods [49, 73, 77]. Increase in precision with increased misclassification can be explained by the expectation of the underlying model that assumes misclassification to be present. Additional analyses showed that improvement in performance of PheLEx over Rekaya was specifically attributed to the use of alternative MCMC algorithm when considering realistic simulations (Fig. 2). Consistent improved performance of PheLEx over Rekaya in identifying misclassified samples from simulated misclassified phenotypes was also observed across differential thresholds on filtering training SNPs (Additional file 1: Text S3).

Improvement in performance was accompanied by a boost in speed for PheLEx (Fig. 2). Results from applying PheLEx and Rekaya to simulation datasets (for the same number of MCMC iterations) were used to track running time for each method. Though accounting for mixed effects due to genetic relatedness/population structure requires additional time, across all simulations PheLEx (median time: 37.2 min) was around 11 times faster than Rekaya (median time: 411.6 min). Running time details for PheLEx-mm and PheLEx-mh are included in Additional file 1 (Text S2).

### Identification of novel GWAS associations by PheLEx misclassification correction

To explore the impact of identifying misclassified samples on association analysis, corrected phenotypes were computed using misclassification probabilities obtained from PheLEx for simulated data. Corrected phenotypes were produced from simulated misclassified phenotypes by switching cases (phenotype = disease) with high misclassification probabilities (determined using misclassification probability threshold *t* defined in the methods section) to controls (phenotype = healthy) using PheLEx. Association analyses were performed between genotypes and (i) simulated true phenotypes (no misclassification), (ii) misclassified phenotypes (%false positives: 1, 3, 5, 8, and 10%), and (iii) PheLEx corrected phenotypes. Resulting *p*-values from each association analysis were used to quantify GWAS performance in detecting true positive SNPs (disease-associated SNPs).

**Fig. 3:**
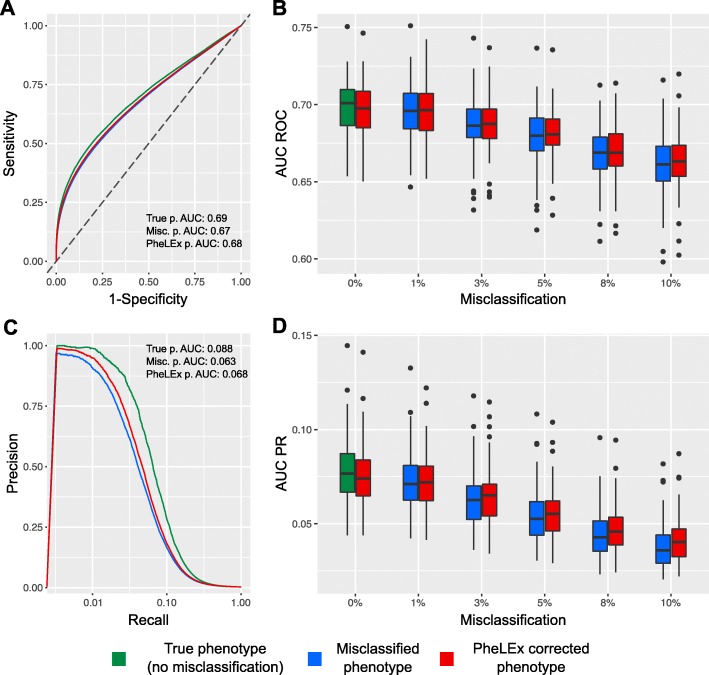
Improvement in GWAS performance via PheLEx in simulations. Results are shown for simulated true phenotype (green; no misclassification), simulated misclassified phenotype (blue), and PheLEx corrected phenotype (red). **a** Receiver Operating Characteristic (ROC) curves are shown with mean Sensitivity (y-axis) and mean 1 - Specificity (x-axis) in identifying disease-associated SNPs using *p*-values obtained from association analyses. **b** Box plots of area under ROC curve (AUC ROC) values (y-axis) are shown across increasing misclassification rates (x-axis). **c** Mean precision (y-axis) over recall (x-axis) curves are shown for identifying disease-associated SNPs using *p*-values obtained from association analyses. **d** Box plots of area under Precision-Recall curve (AUC PR) values (y-axis) are shown across increasing misclassification rates (x-axis)

As expected, with increasing misclassification the AUC ROC values in detecting true positive SNPs for simulated misclassified phenotypes decreased compared to the AUC ROC values for the simulated true phenotype (no misclassification) (Fig. 3 and Table 2). PheLEx corrected phenotypes showed modest improvements in detecting true positive SNPs over misclassified phenotypes across increasing misclassification. AUC PR values for PheLEx corrected phenotypes mirrored these improvements over misclassified phenotypes. At higher misclassification levels, improvement in PheLEx corrected phenotype AUC ROC values and AUC PR values over misclassified phenotype AUC ROC and AUC PR values was higher than at lower misclassification rates. This might be attributed to the lower precision in identifying misclassified samples at low misclassification rates (Fig. 2) as lower precision of switching cases entails loss of true cases (switched to controls by PheLEx) along with misclassified cases in corrected phenotype produced, limiting PheLEx’s ability to recover additional true disease-associated SNPs. However, precision of identifying misclassified individuals increased with misclassification rates, resulting in greater improvements upon misclassified phenotype AUC ROC and AUC PR values at higher misclassification rates.

**Table 2:** Performance evaluation of PheLEx in improving GWAS discovery

Misclassification	Median AUC ROC^a^		Median AUC PR^b^	
	Simulated Misclassified phenotype	PheLEx corrected phenotype	Simulated Misclassified phenotype	PheLEx corrected phenotype
0% (no misclassification)	0.701	0.697	0.0769	0.0739
1%	0.696	0.696	0.0710	0.0720
3%	0.686	0.688	0.0625	0.0651
5%	0.680	0.681	0.0527	0.0553
8%	0.669	0.669	0.0428	0.0458
10%	0.661	0.663	0.0359	0.0403

^a^AUC ROC = Area under Receiver Operating Characteristic curve

^b^AUC PR = Area under Precision Recall curve

**Fig. 4:**
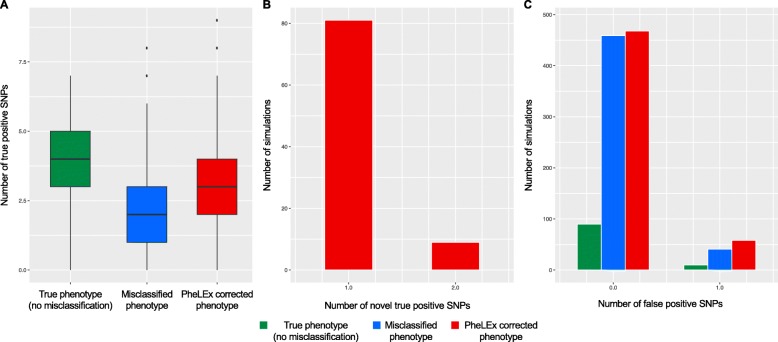
Improving GWAS discovery using PheLEx in simulations. Simulated true phenotype (green; no misclassification), Simulated misclassified phenotype (blue), and PheLEx corrected phenotype (red). **a** Box plots for number of true positive SNPs (disease-associated SNPs) (y-axis) found (using Bonferroni-corrected *p*-value threshold on unadjusted *p*-values) in association analyses with phenotypes (x-axis): simulated true phenotypes (no misclassification), misclassified phenotypes, and PheLEx corrected phenotypes. **b** Bar plot for number of simulations (y-axis) where number of novel true positive SNPs (x-axis) were found (using Bonferroni-corrected *p*-value threshold on unadjusted *p*-values) in association analyses with PheLEx corrected phenotypes. **c** Bar plot for number of simulations (y-axis) where number of false positive SNPs (x-axis) were found (using Bonferroni-corrected *p*-value threshold on unadjusted *p*-values) in association analyses with simulated true phenotypes (no misclassification), misclassified phenotypes, and PheLEx corrected phenotypes

Importantly, when using Bonferroni-corrected *p*-value threshold on unadjusted *p*-values PheLEx identified significant, novel, true positive SNPs defined as disease-associated SNPs that were not statistically significant when analyzing the simulated true phenotypes (no misclassification) or misclassified phenotypes (Fig. 4). These novel discoveries were not accompanied by recovery of large numbers of false positives. PheLEx showed potential to improve discovery of statistically significant disease-associated SNPs (including novel true positive SNPs) with low false positives comparable to those already found in simulated true phenotypes and misclassified phenotypes. In the context of GWAS, this is especially important as any additional loci provide basis for further investigation for their relationship with given phenotype of interest. These results indicate SNP associations and loci discovered by PheLEx are viable hypotheses for making new discoveries in existing GWAS datasets.

### Finding novel associations in real GWAS datasets

PheLEx was applied to UK Biobank GWAS datasets for bipolar disorder (cases = 1177 and controls = 3531) and epilepsy (cases = 3620 and controls = 10,860) to extract misclassification probabilities for the disease phenotypes. Though UK Biobank contained a larger set of individuals for both disease phenotypes, only 1177 and 3620 cases passed the quality control filters for bipolar disorder and epilepsy original phenotypes, respectively. Using a threshold on estimated misclassification probabilities, misclassified cases were identified for each phenotype and their respective phenotypes were switched from case to control, resulting in corrected disease phenotypes. Using these corrected phenotypes, association analyses were performed to investigate genetic associations with the corrected phenotypes. In both analyses, we observed improvement in statistical power of association analysis and identification of new “PheLEx” supplemental candidate loci in GWAS. It is important to note that association analyses results for original bipolar disorder phenotype and original epilepsy phenotype were consistent with previous analyses where UK Biobank genotype datasets were not imputed [89]. In this paper, Manhattan plots shown for both UK Biobank phenotypes were on LD-pruned genotypes with the expected impact on observed peaks in these plots.

#### Bipolar disorder

**Fig. 5:**
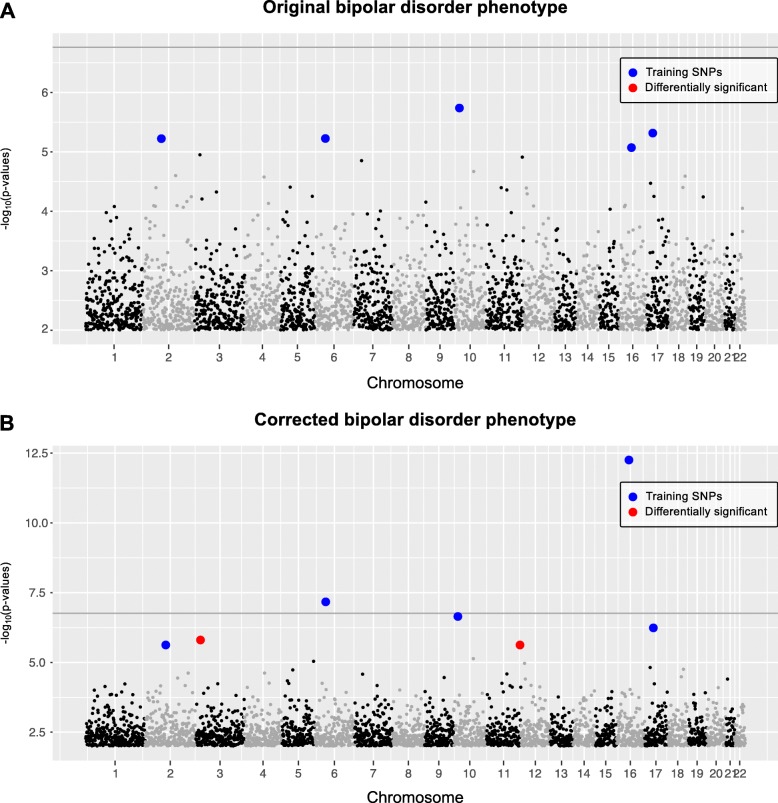
Association analysis of bipolar disorder dataset using PheLEx. Manhattan plots (x-axis: SNP genomic position, y-axis: -log_10_*p*-values of association test > 2) of (**a**) GWAS results for original bipolar disorder phenotype with Bonferroni-corrected *p*-value threshold shown as dark gray line, (**b**) GWAS results for PheLEx corrected bipolar disorder phenotype (where PheLEx-identified cases are switched to controls). Training SNPs used as input for PheLEx are marked in blue whereas differentially significant SNPs are marked in red. Differentially significant SNPs are defined as SNPs not included in training PheLEx that are statistically significant using Benjamini-Hochberg procedure (adjusted *p*-value < 0.1) in association analysis with PheLEx corrected bipolar disorder phenotype and not with original bipolar disorder phenotype. Manhattan plots show linkage disequilibrium (LD) pruned SNPs only

**Fig. 6:**
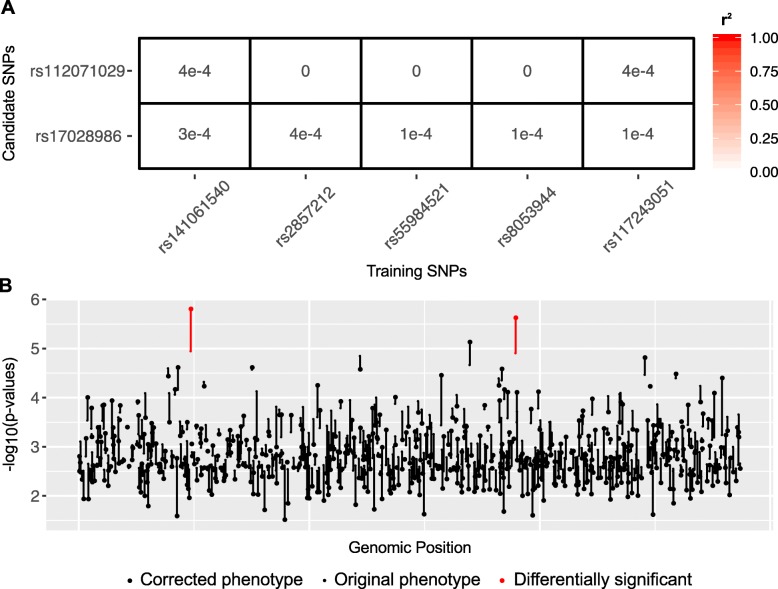
PheLEx discoveries for bipolar disorder phenotype. **a** Heatmap showing r^2^ measure of linkage disequilibrium (LD) computed between the two differentially significant candidate SNPs (rows) identified for corrected bipolar disorder phenotype and training SNPs used as input for PheLEx (columns). Differentially significant SNPs between original and corrected bipolar disorder phenotype with r^2^ < *k*, *k* ~ 1e^− 2^ (not in LD with training SNPs) are identified as PheLEx discoveries. **b** Log transformed *p*-values (y-axis) are reported for a subset of SNPs not in LD with training SNPs plotted in genomic position (x-axis). The small dot for each SNP denotes -log_10_ (*p*-value) obtained from association analysis with original bipolar disorder phenotype and large dot denotes -log_10_ (*p*-value) obtained from association analysis with the PheLEx corrected bipolar disorder phenotype. Differentially significant SNPs (adjusted *p*-value < 0.1) in the PheLEx corrected bipolar disorder phenotype compared to original bipolar disorder phenotype are represented in red

UK Biobank data for original bipolar disorder phenotype (cases = 1177 and controls = 3531) was analyzed using PheLEx and *n* = 54 cases were identified as potentially “misclassified”. “Corrected” bipolar disorder phenotype (cases = 1123 and controls = 3585) was produced where cases identified using PheLEx as “misclassified” were changed to controls. Although, GWAS results with original bipolar disorder phenotype failed to produce any statistically significant SNPs using Bonferroni-corrected *p*-value threshold on unadjusted *p*-values for SNPs or adjusted *p*-values less than 0.1 threshold on *p*-values adjusted using Benjamini-Hochberg procedure (consistent with previous analysis [89]), results from the corrected bipolar disorder phenotype identified candidate SNPs with statistical significance at a Benjamini-Hochberg adjusted *p*-value < 0.1 (Fig. 5). After correction of phenotype, an overall improvement in statistical significance of SNPs was also observed. Apart from training SNPs, SNPs not used in training also gained statistical significance at a Benjamini-Hochberg adjusted *p*-value < 0.1. By computing the r^2^ measure of LD amongst these candidate SNPs and training SNPs, we were able to extract PheLEx discoveries described as candidate SNPs not in LD (r^2^ < *k*, *k* ~ 1e^− 2^) with training SNPs that gained statistical significance at a Benjamini-Hochberg adjusted *p*-value < 0.1 (Fig. 6). Even though most SNPs underwent relatively small changes in their *p*-values (in either direction), PheLEx discoveries experienced a significant boost from their original *p*-values indicating the potential of PheLEx to discover new loci. Identified PheLEx discoveries were not in LD with training SNPs (r^2^ < *k, k* ~ 1e^− 2^) and experienced substantial improvement in statistical significance from original phenotype to PheLEx corrected phenotype (Fig. 6). Table 3 lists details for the PheLEx discoveries including other genes whose SNPs were in LD with them (PheLEx discoveries). One of the PheLEx discoveries was found within the *NTM* gene and was in LD with loci in *OPCML* and *NTM-IT* (Additional file 1: Figure S3). Loci in *NTM* have been previously associated with bipolar disorder and schizophrenia in an independent GWAS [90–92], whereas *OPCML* has also been linked to schizophrenia [91]. The other locus was found in LD with SNPs in *SYN2*, *PPARG,* and *ATG* gene*s. SYN2* has been previously linked with bipolar disorder [93–98] and in GWAS with schizophrenia [99–101], whereas *PPARG* has also been linked to bipolar disorder [102–104] and schizophrenia [105, 106] in other research. *ATG7* has been associated with frontotemporal dementia [107]. Given these previous associations with neurological and psychiatric phenotypes, further investigation and exploration of these PheLEx discoveries is recommended.

**Table 3:** PheLEx discoveries identified for UK Biobank bipolar disorder phenotype

UK Biobank phenotype	SNP	Locus	*p*-value^a^ in original phenotype	*p*-value^a^ in corrected phenotype	r^2b^	Genes^c^	MAF^d^
Bipolar Disorder	rs112071029	11:132129335	1.23e^−5^	6.74e^−7^	4e^− 4^	*NTM, OPCML, NTM-IT, C11orf39*	0.0423
	rs17028986	3:11913699	1.13e^−5^	1.58e^− 6^	3.8e^−4^	*TAMM41, SYN2, PPARG, TIMP4, ATG7, VGLL4*	0.0925
Epilepsy	rs114011598	3:11913699	2.90e^−5^	1.55e^−6^	1e^−4^	*ZLPD1, LOC152225, NXPE3, NFKBIZ*	0.036

^a^ unadjusted *p*-values

^b^ Maximum r^2^ with training SNPs

^c^ Genes with loci in linkage disequilibrium with loci where all annotations were performed using web-resource LDLink

^d^ MAF = Minor Allele Frequency

#### Epilepsy

**Fig. 7:**
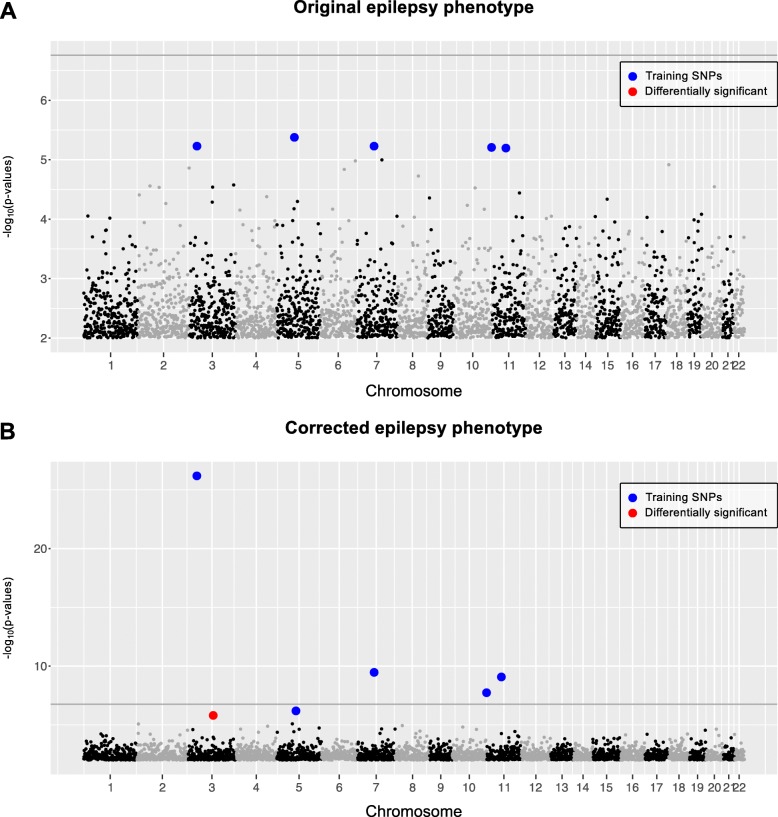
Association analysis of epilepsy dataset using PheLEx. Manhattan plots (x-axis: SNP genomic position, y-axis: -log_10_*p*-values of association test > 2) of (**a**) GWAS results for original epilepsy phenotype with Bonferroni-corrected *p*-value threshold shown as dark gray line, (**b**) GWAS results for PheLEx corrected epilepsy phenotype (where PheLEx-identified cases are switched to controls). Training SNPs used as input for PheLEx are marked in blue whereas differentially significant SNPs are marked in red. Differentially significant SNPs are defined as SNPs not included in training PheLEx that are statistically significant using Benjamini-Hochberg procedure (adjusted *p*-value < 0.1) in association analysis with the PheLEx corrected epilepsy phenotype and not with original epilepsy phenotype. Manhattan plots show linkage disequilibrium (LD) pruned SNPs only

**Fig. 8:**
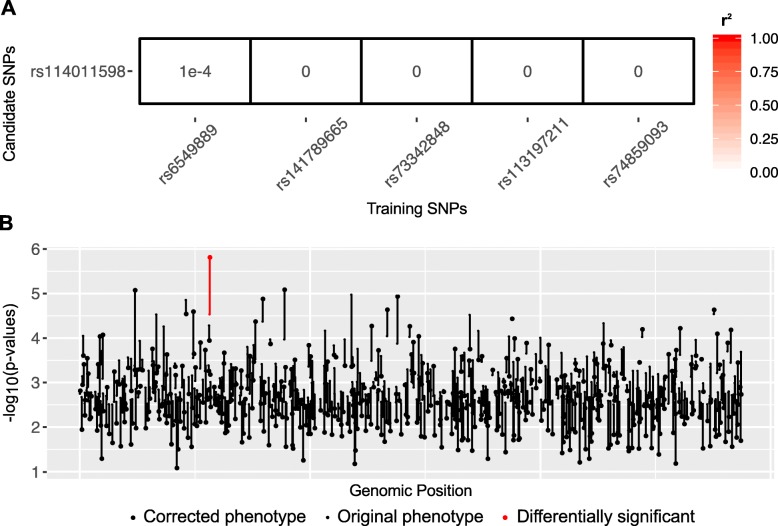
PheLEx discoveries for epilepsy phenotype. **a** Heatmap showing r^2^ measure of linkage disequilibrium (LD) computed between the differentially significant candidate SNP (row) identified for corrected epilepsy phenotype and training SNPs used as input for PheLEx (columns). Differentially significant SNPs with r^2^ < *k*, *k* ~ 1e^− 2^ indicate one PheLEx discovery in corrected epilepsy phenotype that is not in LD with training SNPs. **b** Log transformed *p*-values (y-axis) are reported for a subset of SNPs not in LD with training SNPs plotted in genomic position (x-axis). The small dot for each SNP denotes -log_10_ (*p*-value) in association analysis with original epilepsy phenotype and large dot denotes -log_10_ (*p*-value) in association analysis with the PheLEx corrected epilepsy phenotype. Differentially significant SNPs (adjusted *p*-value < 0.1) in the PheLEx corrected epilepsy phenotype compared to original epilepsy phenotype are represented in red

UK Biobank dataset for epilepsy phenotype (cases = 3620 and controls = 10,860) was analyzed using PheLEx to identify *n* = 395 individuals whose phenotypes might be “misclassified”. These cases were identified as potentially misclassified and their phenotype switched from cases to controls to compute a “corrected” epilepsy phenotype (cases = 3225 and controls = 11,255 controls). GWAS was performed on original epilepsy phenotype and corrected epilepsy phenotype produced by PheLEx to compare results (Fig. 7). Although results of the original analysis were similar to that produced previously for this dataset [89] with no statistically significant SNPs according to Bonferroni-corrected *p*-value threshold or adjusted *p*-values less than 0.1 where *p*-values were adjusted using Benjamini-Hochberg procedure, results from the corrected epilepsy phenotype identified a locus with statistical significance at a Benjamini-Hochberg adjusted *p*-value less than 0.1 and not in LD with training SNPs (Fig. 8). This PheLEx discovery was found in LD with loci within genes *ZPLD1, LOC152225, NXPE3,* and *NFKBIZ* (Table 3, Additional file 1: Figure S3). *ZPLD1* has been associated with onset of sensory disturbances in an independent GWAS [108] and linked to cerebral cavernous malformations [109], which in turn have been linked to high incidence of epilepsy [110]. *NFKBIZ* has been previously associated with amygdala reactivity [111], drug abuse [112], and in GWAS of asthma [113]. Though none of these genes are in the list of known epilepsy genes [114–116], the results suggest a deeper exploration (through fine mapping) of the identified PheLEx discovery may lead to supplemental associations between the epilepsy phenotype and the genomic region.

## Discussion

PheLEx provides two advances when compared to Rekaya, the only existing framework for Bayesian misclassification analysis in GWAS: (i) PheLEx has significantly improved performance for identifying misclassified phenotypes when considering allelic effect sizes in a realistic range observed in GWAS and (ii) PheLEx provides a novel method for identifying potential new phenotype-related loci not detectable with a standard GWAS analysis. The complete PheLEx framework includes the capability to account for differential misclassification (i.e. different rates of misclassification in cases versus controls) while accounting for mixed effects due to genetic relatedness/population structure, a combination which is essential for GWAS analysis. We provide an R package “phelex” [117] to allow application of the entire PheLEx framework for GWAS analysis.

When considering the application of PheLEx in extracting misclassification, there are two aspects of the PheLEx framework that lead to significant improvements in overall performance compared to existing methods [49, 73, 77] in datasets simulated with realistic effect sizes [86, 87]. First, PheLEx includes an Adaptive Metropolis-Hastings step within Gibbs sampling that improves posterior sampling resulting in improved performance in detection of misclassified samples (Fig. 2). Superior performance of PheLEx versus Rekaya in identifying misclassified samples and improving GWAS performance in simulations showed the benefit in using alternative MCMC algorithm (Adaptive Metropolis-Hastings within Gibbs sampling over Gibbs Sampling) along with accounting for mixed effects due to genetic relatedness/population structure. Second, the PheLEx framework uses filtered genotypes as input to prune out SNPs that have a high probability of being uninformative for learning which phenotypes are misclassified, where using more extreme *p*-value thresholds to prune SNPs increases the likelihood of training PheLEx on informative SNPs that will accurately identify misclassified samples (Additional file 1 Text S3). This filtering approach not only provides dramatic savings in terms of computational expense but also improves the accuracy of identifying misclassified samples when considering realistic disease allele effect sizes. Given existing methods [49, 73, 77] do not have acceptable levels of performance for misclassification analysis unless unrealistic allelic effect sizes are considered, PheLEx represents the first Bayesian misclassification that is viable for misclassification analysis for real GWAS.

Though PheLEx showed success in identifying misclassified samples and recovering novel true positive SNPs in simulations, additional considerations should be made in the application of the method and evaluation of PheLEx discoveries. Given the genetic architecture (number of cases = 1000, number of controls = 3000, number of total simulated SNPs = 100,000, and number of simulated disease-associated SNPs = 300) assumed in simulations with realistic effect sizes [86, 87], phenotype heritability values ranged between 0.46–0.57. PheLEx successfully identified misclassified samples in the assumed genetic architecture, however, further investigation is needed to ensure PheLEx’s ability to recover misclassified samples across varied genetic architectures. Moreover, the PheLEx framework makes the (implicit) assumption that cases of misclassification are “random” with respect to the genetic and/or population structure in the data. While we implemented the simulations of GWAS data under this assumption and found PheLEx to have excellent performance for this scenario, there is no question that this assumption won’t hold perfectly in practice (e.g. subgroups of individuals will have higher or lower misclassification rates than others due to shared factors) and depending on how the random misclassification assumption is violated, this could lead to lower power to detect misclassified cases and/or false positives. However, given that in a standard GWAS we will generally not have data available to detect such differences in misclassification rates to correct for them in the analysis framework, a random misclassification assumption is a reasonable first approximation given this lack of information. Finally, we note that PheLEx assumes training SNPs are informative in identifying “misclassified” samples from the given phenotype, where results from this method will not be reliable for datasets where this assumption is not valid. Hence, caution is advised in identification and interpretation of misclassified samples as well as interpretation of any resulting PheLEx driven discoveries. Still, as demonstrated by the analysis of the UK Biobank bipolar disorder phenotype, it is possible for PheLEx to recover supplemental candidate loci for which there is validation evidence (e.g. *NTM* which was previously associated with bipolar disorder in GWAS data independent of the data analyzed from the UK Biobank [90]) indicating the approach has the ability to recover known associations. This supports the assertion that conservatively identified and interpreted PheLEx supplemental candidate loci can potentially provide additional exploratory value beyond candidate loci identified by traditional GWAS analysis.

Considered more broadly, the PheLEx framework is addressing a specific problem of misclassification of disease phenotypes in GWAS that is really a function of the overlapping issues of measurement error and incomplete understanding of disease etiology. These can manifest in a number of ways, including misclassification due to (i) disease similarity and inaccurately measured current diagnosis protocols [57, 58, 118] and (ii) heterogeneous diseases or disease complexes defined as the “same” disease with a single diagnosis protocol [119–122]. Though misclassification may be modelled as a function of genetic similarity across samples and/or other covariates (e.g. disease comorbidity, race, and socioeconomic status), PheLEx framework is agnostic to the cause of misclassification and rather assumes that the underlying genetics can be leveraged to provide an accurate assessment of misclassification, regardless of cause, and is of value whether used purely for identifying misclassification or potential loci for phenotypic associations. Hence, samples marked as “misclassified” using PheLEx may be a result of the underlying heterogeneity of the disease complex (and not misclassification) whereas the “corrected phenotype” may define a closely related phenotype, secondary phenotype or subtype of the disease. For misclassification, there is clear value in identifying healthy individuals who were misdiagnosed such that PheLEx presents an opportunity to identify “false cases” and investigate basis of their misclassification, which may relate to diagnosis error, disease subtypes or differential patterns in diagnostic measurements.

## Conclusion

Keeping an eye on its limitations, PheLEx has promise as a novel analysis methodology for identifying exploratory loci in GWAS that can be applied to reanalyze existing GWAS data.

Accounting for mixed effects due to genetic relatedness/population structure and use of efficient MCMC algorithm allows PheLEx to leverage association between phenotype and genotypes to extract misclassification from existing phenotypes. By defining more tractable GWAS phenotypes, PheLEx can boost power of association analyses and identify new loci of interest.

## Methods

### The PheLEx framework

#### PheLEx framework overview

The PheLEx analysis framework (Fig. 1), available as a function in R package “phelex” [117], is designed to identify misclassified disease phenotypes using GWAS data. PheLEx requires three inputs: (i) a sample of *n* observed disease phenotypes *Y*, (ii) a set of genotypes (SNP) identified as having a strong association with phenotype in a GWAS analysis *X,* and (iii) genetic relatedness or kinship matrix *A* (which may reflect genetic relatedness and/or population structure and can be estimated from the genotype data of a GWAS). Using SNPs with strong associations with the disease phenotype as PheLEx input provides a dual advantage of (i) making the framework applicable to real GWAS data that include genotypes on the scale of hundreds of thousands of SNPs (where simultaneously analyzing all SNPs in the underlying modeling framework would be computationally prohibitive) and (ii) filtering of non-informative GWAS genotypes from the PheLEx training input that improves the performance of the method (since the stronger the predictive ability of the training SNPs, the greater the ability to detect a case of misclassification). The phenotype-associated SNPs used as input to PheLEx can be selected using any GWAS statistical tests of association with some threshold for selection *p*, which is a parameter of the framework set by the user (see subsections below for discussion of approaches for statistical testing and values of *p* used for the current work).

The inputs to PheLEx are used in combination with the underlying PheLEx model to identify cases of misclassification. The PheLEx model of misclassification is a hierarchical probabilistic model that includes parameters for true positive (*α*) and false positive probabilities (*λ*) of misclassification in the observed disease phenotype (see subsection below). For inference, a Bayesian approach is applied making use of an MCMC algorithm, specifically a Gibbs Sampler that includes an Adaptive Metropolis Hastings step. The Adaptive Metropolis Hastings within Gibbs sampler is used to estimate the posterior probability that the phenotype of a sample is misclassified. This approach therefore produces a posterior probability of misclassification for each sample, where a threshold *t* is used to make a decision about which samples are misclassified. The parameter *t* is set by the user to control how conservative the framework is when determining cases of misclassification (see subsections below for discussion of values of *t* used for the current work).

Samples determined to be misclassified by the PheLEx framework could be the objective of a study (e.g. considered for removal from an analysis or investigated for possible sources of misclassification in defining phenotypes). These cases of misclassification could also be used within an additional application of PheLEx by “correcting” such cases (i.e. switching misclassified cases to controls and/or misclassified controls to cases). The set of phenotypes including these PheLEx corrections can then be used to perform association analysis in a GWAS, providing the potential to discover additional genotypes associated with the phenotype that were not strongly associated when considering (uncorrected) misclassified samples. We note that such new associated genotypes should be considered with caution since they involve possible errors in the identification of misclassified phenotypes and are best used as a starting point for additional investigation.

#### PheLEx framework assumptions

The PheLEx misclassification framework makes a number of strong assumptions about the structure of the data that can impact the misclassification inferences. The first major assumption is that only a small fraction of the GWAS phenotype is misclassified, where large estimates of misclassification could produce spurious associations that could lead to false positive assessments of misclassified phenotypes. The second major assumption is that there are a number of phenotype-associated genotypes (SNPs) with strong association with the phenotype that can be identified in a GWAS analysis and used as input to the algorithm (i.e. statistical significance of association between the SNP and phenotype passes the filter parameter *p*). Since the ability to identify cases of misclassification improves with the phenotype predictive ability of the set of SNPs selected as input, if the selected SNPs have weak associations we would not expect the framework to return any samples with a high posterior probability of being classified. Conversely, if a large number of SNPs identified with strong associations with the phenotype that represent false positives are selected as input, this could result in misclassification false positives. A third major assumption implicit to the PheLEx underlying misclassification model is that the misclassified phenotypes are random with respect to the underlying genetics. If this is not the case, the result could be lower power of the framework to detect cases of misclassification or if cases of misclassification are correlated with some factor that is also correlated with sets of genotypes, the result could be false positives. A fourth major assumption of the framework is that the underlying PheLEx probability model provides a reasonable approximation to the genetic and the misclassification structure in the GWAS data (e.g. that the overall probability of misclassification can be captured with a latent variable representation and the parameters *α* and *λ*) where a poor approximating model and/or an MCMC algorithm that results in poor inference given the observed data can result in lower power and/or false positives. A fifth major assumption is that parameter *t* is not set so low to allow lots of false positive assessments of misclassification.

We note that while all of these are strong assumptions of the PheLEx framework that any user should be both aware of and incorporate into their assessments of the output given the unknowns in their GWAS data, these are not uncommonly strong assumptions. While the framework does make an assumption that there is a small proportion of misclassified cases in the given phenotype, this is reasonable for numerous disease phenotypes where established diagnosis criteria may lead to a small fraction of misclassifications and is not expected to be so poorly defined that cases of misclassification are in the majority (and in turn drive large numbers of false positives when analyzing SNP associations). The assumption that there are strong SNP associations with the phenotype appears to be reasonable for a large number of diseases analyzed with GWAS. Given that these SNPs are identified with a stringent enough cutoff (i.e. the parameter *p*) using a GWAS analysis approach that accurately corrects for confounding factors that may produce false positives is a core assumption of the GWAS analysis approaches. While cases of misclassification are almost certainly not random and could, in theory, be detected with more complete data (e.g. a rich clinical record or a family history for each individual in the study), data required to assess such patterns of misclassification is often not available. Thus, it is reasonable to assume misclassification is random as a first approximation, particularly given the information required to make stronger inferences on what is driving the patterns of misclassification is typically missing/uncertain in data provided. Although applying a single probabilistic model of the genetics, population structure, and misclassification is also a strong assumption, it does not seem overly strong given the unknown structure present in GWAS data, particularly because we expect better inference results when assuming a relatively simple model that limits the number of parameters. Finally, it is assumed in practice that the user will set a relatively conservative value for the parameter *t* such that only cases with a strong posterior probability of being misclassified are designated as such, where setting a conservative value for this parameter also seems a reasonable policy in general when applying the PheLEx framework. However, setting the parameter *t* (or *p*) is ultimately up to the user, where setting a liberal threshold for these parameters is not necessarily problematic as long as the user is aware that the more liberal these parameters are set, the higher the probability of false positives, such that the results should be considered with greater caution.

#### PheLEx framework Bayesian vs. frequentist considerations

The PheLEx framework can be made into a fully Bayesian approach by applying a Bayesian GWAS analysis used to select the set of input SNPs *X*. For example, a fully Bayesian approach that makes use of the same mixed model incorporated into the PheLEx hierarchical model could be applied by application of a Bayesian Linear Mixed Model (BLMM) to GWAS data and subsequent use of Bayes factor to assess associations, where the parameter *p* is set for an appropriate cutoff for the Bayes factor. This possibility was explored by implementing a BLMM GWAS analysis as the initial step using the R package “GridLMM” [123], and the option of applying such an approach to make the framework completely Bayesian could be implemented by the user. To note however, (as expected) high correlations (e.g. in the range 0.95–0.97, results not shown) were observed when comparing the ranking of SNP associations as assessed using Bayes factor values from a BLMM analysis versus *p*-values returned from a linear mixed model analysis for simulated GWAS data. A qualitatively different result is therefore not expected when employing a fully Bayesian approach. In the current study a frequentist testing approach was applied to select the set of input SNPs (training SNPs) and to analyze GWAS results after “correcting” misclassified phenotypes identified with the PheLEx framework. This strategy was used because the vast majority of GWAS analyses make use of *p*-values to identify associated SNPs, such that using this approach (and setting a corresponding *p*-value threshold for *p*) is a more natural fit given usual GWAS workflow and therefore in a sense more “natural” for a GWAS practitioner.

#### The PheLEx framework misclassification model

In the absence of misclassification in phenotype, the relationship between genotypes matrix *X* (composed of *m* SNPs) and observed phenotype *Y* (for *n* individuals) can be stated as,

$$ \Pr \left(Y|\beta, u\right)\propto \prod \limits_{i=1}^n\sigma {\left(\beta, u\right)}^{Y_i}{\left(1-\sigma \left(\beta, u\right)\right)}^{1-{Y}_i} $$

with

$$ \mathrm{Y}=\left[{\mathrm{Y}}_1,\dots, {\mathrm{Y}}_{\mathrm{n}}\right] $$

$$ \mathrm{X}=\left[{\mathrm{X}}_1,\dots, {\mathrm{X}}_{\mathrm{m}}\right] $$

where:

$$ \Pr\ \left(Y=1\vert \beta, u\right)=\sigma\ \left(\beta X+u+\epsilon \right) $$

$$ u= MultivariateNormal\left(0,{\sigma}_u^2A\right) $$

and where *X*_*j*_ is a genotype vector for SNP *j, σ(β, u)* is a function of SNP effects *β* and random effects *u*, *σ*_*u*_^*2*^ is the variance parameter, and *A* is the genetic relatedness matrix.

In presence of misclassification, the relationship between *X* and *Y* is modeled in PheLEx using a hierarchical Bayesian latent variable model, where the relationship between *X* and *Y* is intermediated through (i) a latent variable representing true phenotype *Y′*, (ii) the false positive rate in phenotype (*λ*) representing rate of true controls recorded as cases, and (iii) the true positive rate in phenotype (*α*) representing rate of true cases recorded as cases. With these additional assumptions, the relationship between genotypes *X* and true phenotype *Y′* can be stated as:

1 $$ \Pr \left({Y}^{\prime }|\beta, u\right)\propto {\prod}_{i=1}^n\sigma {\left(\beta, u\right)}^{Y{\prime}_i}{\left(1-\sigma \left(\beta, u\right)\right)}^{\left(1-{Y}_i\prime \right)} $$

with the resulting likelihood of observing the data (*X* and *Y*) given unknown parameters:

$$ \Pr \left(Y|{Y}^{\prime },\alpha, \lambda, \beta, u,{\sigma}_u^2\right)\propto \Pr \left(Y|\lambda, {Y}^{\prime }=0\right)\Pr \left({Y}^{\prime }=0|\beta, u\right)+\Pr \left(Y|\alpha, {Y}^{\prime }=1\right)\Pr \left({Y}^{\prime }=1|\beta, u\right) $$

2 $$ \Pr \left(Y|{Y}^{\prime },\alpha, \lambda, \beta, u,{\sigma}_u^2\right)\propto {\prod}_{i=1}^n{\left[\lambda \left(1-\sigma \left(\beta, u\right)\right)+\alpha \left(\sigma \left(\beta, u\right)\right)\right]}^{Y_i}{\left[\left(1-\lambda \right)\left(1-\sigma \left(\beta, u\right)\right)+\left(1-\alpha \right)\left(\sigma \left(\beta, u\right)\right)\right]}^{\left(1-{Y}_i\right)} $$

For this model, the posterior probability is:

3 $$ \Pr \left({Y}^{\prime },\alpha, \lambda, \beta, u,{\sigma}_u^2|Y\right)\propto {\prod}_{i=1}^n{\left[\lambda \left(1-\sigma \left(\beta, u\right)\right)+\alpha \left(\sigma \left(\beta, u\right)\right)\right]}^{Y_{i.}}{\left[\left(1-\lambda \right)\left(1-\sigma \left(\beta, u\right)\right)+\left(1-\alpha \right)\sigma \left(\beta, u\right)\right]}^{\left(1-{Y}_i\right)}\Pr \left(\alpha \right)\Pr \left(\lambda \right)\Pr \left(\beta \right)\Pr \left(u|{\sigma_u}^2\right)\Pr \left({\sigma_u}^2\right) $$

For identification of cases that are misclassified in PheLEx, the interest is not in the full posterior but rather in the marginal posterior for an individual conditional on the state of the phenotype and that the latent variable is in the opposite state:

4 $$ \mathrm{Misclassification}\ \mathrm{in}\ \mathrm{cases}\sim \mathrm{Binomial}\ \left({n}_1,\Pr \left(Y^{\prime }=0|Y=1,\alpha, \lambda, \beta, u\right)\right) $$

5 $$ \mathrm{Misclassification}\ \mathrm{in}\ \mathrm{controls}\sim \mathrm{Binomial}\ \left({n}_2,\Pr \left(Y^{\prime }=1|Y=0,\alpha, \lambda, \beta, u\right)\right) $$

The posterior parameter probabilities in these eqs. (4–5) are determined using the PheLEx MCMC algorithm (see next subsection). At each iteration, the marginal posterior probabilities of being misclassified are calculated eqs. (10–11) and an individual is determined to be misclassified using eqs. 4–5. Across iterations, average misclassification probability of each individual is computed as the number of times the individual was marked as misclassified divided by the total number of iterations. An individual is determined to be misclassified if the average misclassification probability exceeds the value *t* (set by the user).

#### The PheLEx MCMC algorithm

The PheLEx MCMC is an Adaptive Metropolis-Hastings within a Gibbs Sampler to estimate model parameters. Parameters (*α, λ,* and *β*) are sampled with Adaptive Metropolis-Hastings algorithm using the following steps:

1. Initialize random starting values for parameters *α, λ,* and *β* using the respective distributions to sample starting values. Set *u* as a zero vector and *σ*_*u*_^*2*^ = 0.1
2. Define the proposal
  - Sample values for *α* and *λ* using truncated normal distribution
  - Sample values for *β* using normal distribution
3. Calculate posterior probabilities from the current parameter values and proposed parameter values
  - Compute *σ(β, u)* for current parameter values and proposed parameter values
  - Compute posterior for current values and proposed parameter values using Eq. 3:
    $$ \Pr \left(Y^{\prime },\alpha, \lambda, \beta, u|Y\right)\propto {\prod}_{i=1}^n{\left[\lambda\ \left(1-\sigma \left(\beta, u\right)\right)+\alpha\ \left(\sigma \left(\beta, u\right)\right)\right]}^{Y_{i.}}{\left[\ \left(1-\lambda \right)\ \left(1-\sigma \left(\beta, u\right)\right)+\left(1-\alpha \right)\left(\sigma \left(\beta, u\right)\right)\right]}^{\left(1-{Y}_i\right)}\ \Pr \left(\alpha \right)\ \mathit{\Pr}\left(\lambda \right)\Pr \left(\beta \right) $$
  - Update values for parameters with proposed parameter values with acceptance probability $p=\frac{Posterior\ probability\ with\ proposed\ values}{Posterior\ probability\ with\ current\ values}$

Parameters *σ*_*u*_^*2*^ and *u* are estimated in the following Gibbs step using conditional probability distributions for each parameter as defined in previous literature [124–126]. At each iteration, misclassification in each sample of the phenotype is also estimated.

1. Given *l*_*i*_ = *X*_*i*_*β* + *u*_*i*_(6)
2. $$ {u}_i\mid \beta, {u}_{-i},l,{\sigma_u}^2,Y\sim N\ \Big(\hat{u_i},\left(1+{c}_{ii}\gamma \Big){}^{-1}\right)(7)\ \mathrm{where}, $$
  - $$ \hat{u_i}={\left(1+{c}_{ii}\gamma \right)}^{-1}\left(\left({l}_i- X\beta \right)-\gamma {c}_{i,-i}{u}_i\right)(8) $$
  - *c*_*ii*_ = *ith diagonal element of A*^−1^
  - *c*_*i*, − *i*_ = *row i of A*^−1^ *with element i removed*
  - *u*_−*i*_ = *vector u with element i removed*
  - *γ* = (*σ*_*u*_^2^)^−1^
3. $$ \Pr \left({\sigma_u}^2|\beta, u,l,Y^{\prime}\right)\propto {\left({\sigma_u}^2\right)}^{-\left(\frac{q}{2}\right)}\mathit{\exp}\ \left(-\frac{u^{\prime }{A}^{-1}u}{2{\sigma_u}^2}\right)1\left(0,{\sigma_{umax}}^2\right)\kern1.75em (9) $$
  - *σ*_*umax*_^2^ = 100
4. Estimate misclassified phenotypes using
  - Misclassification in cases ~ Binomial (*n*_*1*_, Pr(*Y’*=0|*Y*=1, *α, λ, β, u*)) (4)
    i. $$ \Pr \left({Y}_i^{\prime }=0|{Y}_i=1,\alpha, \lambda, \beta, {u}_i\right)=\frac{1}{1+\frac{\alpha\ \sigma \left(\ \beta, {u}_i\right)}{\lambda\ \left(1-\sigma \left(\beta, {u}_i\right)\right)}}(10) $$
    ii. *n*_*1*_ = total number of cases
  - Misclassification in controls ~ Binomial (*n*_*2*_, Pr(*Y’*=1|*Y*=0, *α, λ, β, u*))) (5)
    i. $$ \Pr \left({Y}_i^{\prime }=1|{Y}_i=0,\alpha, \lambda, \beta, {u}_i\right)=\frac{1}{1+\frac{\left(1-\lambda \right)\left(1-\sigma \left(\ \beta, {u}_i\right)\right)}{\left(1-\alpha \right)\left(\sigma \left(\beta, {u}_i\right)\right)}}\kern1.75em (11) $$
    ii. *n*_*2*_ = total number of controls

At each iteration, the probabilities in eqs. (10–11) are then used to determine whether an individual is considered to be misclassified through eqs. (4–5). Across iterations, average misclassification probability of each individual is computed as the number of times the individual was marked as misclassified divided by the total number of iterations. An individual is determined to be misclassified if the average misclassification probability exceeds the cutoff *t*.

#### PheLEx framework priors and identifiability considerations

Without placing priors on the misclassification parameters, the full PheLEx model likelihood and therefore posterior is unidentified. Such identifiability issues in Bayesian mixture models are well appreciated due to the “label switching problem” [127–129]. Following the approaches of others when using mixture models for Bayesian inference [128, 129], we introduce identifiability constraints to restrict the parameter space by defining *logit*(P(*Y* = 1|*Y′* = 1)) > *logit*(P(*Y* = 1|*Y′* = 0)) where *Y* is the observed (misclassified) phenotype and *Y′* is the latent true phenotype. This is implemented in the PheLEx framework by using informed priors where the prior on true positive probability *α* parameter follows a Beta distribution Pr(*α*) ~ Beta(10, 1) whereas the prior on false positive probability *λ* parameter follows a Beta distribution Pr(*λ*) ~ Beta(1, 1). For the prior on *α* this places a high probability that controls in a GWAS are not misclassified, which seems a reasonable assumption for most GWAS studies where to be considered a case of disease, an individual needs to adhere to a relatively complex set of criteria. In contrast, for the case misclassification rate, we assume a flat (uniform) prior, such that we are not making a strong prior assumption on the probability that a case is misclassified. We additionally note that we assume a flat prior on variance parameter *σ*_*u*_^*2*^ [125, 126] and normal prior Pr(*β*) ~ N(0, 1) on the distribution of SNP effects with true genetic associations, where this latter assumption seems justified given estimates of SNP associations in GWAS [83–87].

The advantage of setting a flat prior on *λ* (i.e. the probability of a true control being misclassified as a case) is such prior does not have a strong impact on the inference that an individual is misclassified, where for simulated data, we found this assumption lead to excellent performance of the PheLEx framework and reasonable outcomes when analyzing real GWAS data (see Results section). However, a disadvantage of a flat prior on *λ* is the joint marginal posterior for *α* and *λ* is bimodal, where there is a second mode that represents a “label switch” such that the majority of cases and controls are both considered to be misclassified. While the posterior probability of this mode is low enough that it does not dramatically impact performance of the overall framework, it does have the disadvantage that it is not intuitively “interpretable” (i.e. in GWAS we don’t generally assume almost all of the cases and controls are mislabeled). Given that this is a true label switch, a more complete sampling of the posterior using efficient proposals or adaptation mechanisms such as simulated tempering [130] followed by traditional (i.e. median) Bayesian inference of parameters would not produce better performance because the impact of the modes would be to “cancel” one another. There are two possible solutions to this issue: the first is to apply stronger priors on the model hyperparameters to assure the posterior is unimodal (e.g. a non-flat prior on *λ*) and the second is to “throw-out” posterior probability estimates returned by the algorithm that are driven by the “switch” mode, which are easily identified by looking at the values of *α* and *λ*. We apply the latter approach for the current study, which while admittedly is a heuristic (and therefore not a true Bayesian approach), we found that applying this approach with an uninformative flat prior on *λ* produces excellent performance when assessing simulated data (see Results section), where a framework that performs well in practice was our goal when developing this method.

#### PheLEx framework inference

When running the PheLEx MCMC, the variance for jumping distribution of effect sizes is adjusted across iterations to maintain acceptance ratio for MCMC chains around 0.2 using established methods [131]. For the simulation and real data analyses, an acceptance rate of 0.2 was used [132] and the algorithm was run on each dataset for 100,000 iterations with a burn-in of 20,000 iterations. At each iteration, estimates for each parameter(*α*, *λ*, *β,* and *u*) were used to calculate misclassification probability for each sample in the phenotype, where the average misclassification probability for each sample was computed by summing over the number of times a sample was deemed as misclassified (Step 4) and dividing by total number of iterations. To assess convergence, Geweke’s convergence diagnostic [133] was applied, where a convergence of parameters is indicated if the Geweke z-scores lie within the 95% confidence interval (− 1.96 to 1.96). While we found that the heavy majority of the chains we ran for simulated and real data converged with the median z-score across parameter estimates close to 0, a practical disadvantage introduced by bimodal structure of the joint marginal posterior of *α* and *λ* is chains do not always converge. We therefore suggest running multiple chains and using chains that converge as indicated by Geweke’s diagnostic [133].

### The Rekaya misclassification framework and variants of PheLEx

To provide a baseline for assessing performance of the PheLEx framework, we compared PheLEx to the only existing misclassification framework designed for the analysis of GWAS data [49, 73, 77], which we have denoted as Rekaya. Rekaya used a full Gibbs sampler to estimate misclassification rates (false positive rate and false negative rate) and identify misclassified samples from GWAS datasets, without accounting for random effects due to genetic relatedness/population structure. In addition to the main comparison of PheLEx and Rekaya [49, 73, 77] we also implemented two variants of PheLEx to determine the impact of the two major differences between PheLEx and Rekaya: (i) PheLEx-mm (PheLEx −/minus mixed model): includes an Adaptive Metropolis-Hastings step in the MCMC algorithm not present in Rekaya Gibbs sampler and excludes the mixed model that accounts for genetic relatedness/population structure and (ii) PheLEx-mh (PheLEx −/minus Metropolis Hastings): includes a full mixed model that can account for genetic relatedness/population structure and excludes the Adaptive Metropolis-Hastings step in the Gibbs sampler. Implementation steps and parameter initialization for the published Rekaya and the two variants of PheLEx, which either exclude the Adaptive Metropolis-Hastings step (PheLEx-mh) or exclude the mixed model (PheLEx-mm) are included in Additional file 1: Text S4.

### Simulation study

#### Simulation datasets

Two strategies were employed to simulate data for assessing framework performance. For the first strategy, datasets were simulated to allow a comparison of the variant of PheLEx without the mixed model (PheLEx-mm) to Rekaya to provide fair assessment of the performance impact of the Adaptive Metropolis-Hastings step in PheLEx compared to Rekaya when considering a GWAS scenario where there is no genetic relatedness/population structure. For these simulations, genotypes were simulated using “simulateGenotypes” function from R package PhenotypeSimulator [134] for 2000 samples and 10,000 independent SNPs. Minor allele frequency (MAF) for simulated SNPs was sampled from multinomial distribution with means 0.1, 0.2, and 0.4 (default parameters for “simulateGenotypes” function). One hundred true disease phenotypes (*Y′*) were simulated with 30 randomly selected simulated genotypes using the following relationship:

12 $$ \Pr\ \left(Y^{\prime }=1|\beta \right)=\sigma \left(\beta X+\epsilon \right) $$

13 $$ \mathrm{where}\kern0.50em \epsilon \sim N\ \left(0,1\right),\beta \sim N\ \left(2,0.3\right) $$

Here, *σ* is a probit link function, *β* are fixed effect sizes of disease-associated SNPs *X,* and *ε* represents noise. Thirty SNPs were randomly selected to be disease-associated SNPs *X* for all true phenotypes *Y′*. Fixed effects *β* for *X* were sampled for each disease phenotype separately from normal distribution with mean and variance parameter values stated above. For each simulated true disease phenotype *Y′* (1000 cases and 1000 controls), differential misclassification was introduced at varying degrees by switching a fraction of randomly selected controls to cases. Fraction of controls switched to cases varied from 5, 10, 20, 30, and 40% representing increasing rates of misclassification in “observed phenotype” denoted as *Y*. Resulting datasets consisted of 100 datasets for each misclassification rate (5, 10, 20, 30, and 40%).

For the second strategy, data were simulated for the comparisons of PheLEx and Rekaya when including genetic relatedness/population structure. For these simulations, genotypes were simulated using simulateGenotypes function from the R package “PhenotypeSimulator” [134] for 10,000 samples and 100,000 independent SNPs, i.e. SNPs not in LD. MAF for simulated SNPs was sampled from uniform distribution with range between 0 and 0.5. One hundred true disease phenotypes for the population (*Y*_*pop*_*’*) with disease prevalence in range 0.1–0.5 were simulated for *n* = 10,000 samples using the following relationship:

14 $$ \Pr \left({Y}_{pop}^{\prime }=1|\beta, u\right)=\sigma \left(\beta X+u+\epsilon \right) $$

$$ \mathrm{where}\varepsilon \sim N\left(0,1\right),\beta \sim N\left(0,{\sigma}_g^2{\left[2f\left(1-f\right)\right]}^{\alpha}\right),u\sim N\left(0,{\sigma_u}^2A\right),{\sigma_u}^2=2,{\sigma}_g^2=.1,\alpha =-0.38 $$

Here, *σ* is a probit link function, *β* are fixed effect sizes of disease-associated SNPs *X*, *u* is a simulated random effects vector, *ε* represents noise and *f* is the MAF of disease-associated SNPs. *A* is a square genetic relatedness matrix (*n* = 10,000) computed using getKinship function from R package “PhenotypeSimulator”. Random effects vector *u* was simulated from multivariate normal distribution using function mvrnorm from R package “MASS” with variance parameter *σ*_*u*_^*2*^, relatedness matrix *A* and mean as zero vector. This configuration of simulated GWAS datasets was in stark contrast to simulated data analyzed previously, where 150 out of 1000 simulated SNPs were associated with true disease phenotype with unrealistic maximum genotype-specific disease odds-ratio in range 4–10 in each dataset [49, 77].

Three hundred SNPs were randomly selected to be disease-associated SNPs *X* for all simulated true population phenotypes *Y*_*pop*_*’* (*n =* 10,000 samples). The same computed random effects vector was used to simulate all true population disease phenotypes *Y*_*pop*_*’*. Fixed effects *β* for *X* were sampled for each simulation from a normal distribution with mean and variance parameter values stated above, following a previously suggested model [86, 135, 136], whereas percentage phenotypic variance explained by each disease-associated SNP (*X*) was calculated using the relationship [137]:

$$ \mathrm{Phenotypic}\ \mathrm{variance}\ \mathrm{explained}\ \mathrm{by}\ \mathrm{SNP}\ l=\frac{Var\ \left({X}_l{\beta}_l\right)}{Var\ \left(\ \left({\sum}_{j=1}^m{X}_j{\beta}_j\right)+u+\epsilon\ \right)} $$

The genetic model with 300 disease-associated SNPs with realistic effect sizes out of total 100,000 SNPs preserves the characteristic sparsity of “true signal” in GWAS datasets, with phenotypic variance explained by each SNP in the empirically observed range of 1e^− 9^%– 3%. Overall, the number of SNPs, number of disease-associated SNPs, phenotype heritability values, and simulated effect sizes were set in accordance with precedence in literature [86, 87, 138–140].

From each of the 100 simulated true population phenotypes *Y*_*pop*_*’*, a total of 1000 cases and 3000 controls were sampled to produce simulated true phenotypes *Y′* with *n* = 4000 samples for GWAS analysis*.* For each GWAS, differential misclassification was introduced to alter the simulated true phenotype *Y′* at varying degrees by switching a fraction (1, 3, 5, 8, and 10%) of randomly selected controls to cases, resulting in 1, 3, 5, 8, and 10% false positives in “observed phenotype” or “simulated misclassified phenotype” denoted as *Y.* The overall simulation analysis therefore considered 100 simulated GWAS datasets with true phenotypes (no misclassification) and 100 simulated GWAS datasets with misclassified phenotypes at each misclassification rate, all simulated with mixed effects of genetic relatedness/population structure.

#### Comparison of PheLEx and Rekaya

Misclassification extraction methods PheLEx and Rekaya were applied to the simulation datasets to identify misclassified samples from each simulated misclassified phenotype. We performed standard GWAS analysis between each simulated misclassified phenotype against genotype data and used the Bonferroni-corrected genome-wide *p*-value threshold (*p* < 10^–6.3^) to filter out potentially uninformative SNPs. For PheLEx and Rekaya, resulting input genotypes matrix (training SNPs) contained SNPs whose unadjusted *p*-values were lower than Bonferroni-corrected genome-wide threshold for the dataset. For all analyses with PheLEx, inputs included simulated misclassified phenotype, training SNPs, and a genetic relatedness matrix computed using R function getKinship on all SNPs for that dataset with MAF > 5% [134]. Input for Rekaya only included the training SNPs and each misclassified phenotype. For each simulated misclassified phenotype, training SNPs (along with other input information) were processed through PheLEx and Rekaya for 100,000 iterations, and misclassification probabilities for each case and control in simulated misclassified phenotype was returned as output. For all analyses, a misclassification probability threshold (*t =* 99th percentile of misclassification probabilities estimated for cases in *Y*) was selected, where all cases (i.e. *Y* = 1) with misclassification probabilities greater than *t* were marked as misclassified. Precision, recall/true positive rate, and false positive rate were calculated for misclassified cases identified by PheLEx in simulations and compared to Rekaya’s performance.

Misclassified samples/cases found in simulations (where training SNPs were filtered using *p* < 10^–6.3^ and misclassification probability threshold *t* = 99th percentile) by PheLEx were further used to create respective corrected phenotypes by switching phenotype of misclassified cases from case to control. Association analyses were performed with corrected phenotypes produced by PheLEx against genotype data. Performance metrics (i.e. precision, recall/true positive rate, and false positive rate) were computed on resulting *p*-values produced from association analyses and compared between phenotypes: simulated true phenotypes, simulated misclassified phenotypes, and PheLEx corrected phenotypes. Additional analyses were performed to observe the impact of varying (i) *p*-value threshold for filtering training SNPs (*p* < 10^− 4^, *p* < 10^− 5^, and *p* < 10^–6.3^) and (ii) misclassification probability threshold *t* (*t* = 99th percentile, *t* = 95th percentile, *t* = 90th percentile, *t* = 85th percentile, *t* = 80th percentile, and *t* = 75th percentile) on method’s performance to identify misclassified samples and disease-associated SNPs (Additional file 1: Text S3).

Performance for each method applied was measured by each misclassification extraction method’s ability to identify misclassified cases. Using average misclassification probabilities estimated by PheLEx and Rekaya over 100 simulated datasets at each misclassification level (1 to 10%), performance metrics such as recall/true positive rates, false positive rates, and precision were calculated for each method. For decreasing threshold values in range 0.0–1.0, cases with misclassification probability higher than threshold were marked as misclassified by the method. Recall/true positive rate was calculated as the fraction of correctly identified misclassified cases out of all misclassified cases in the dataset and false positive rate was calculated as fraction of true cases labeled as misclassified out of all true cases. Precision was calculated as the fraction of correctly identified misclassified cases in the set of misclassified cases marked by each method. For visualization of ROC curves, mean true positive rate across 100 simulations at each false positive rate value was calculated per method. Similarly, for PR curves, mean precision across 100 simulations at each recall value was calculated per method. AUC ROC and AUC PR values were computed by calculating the area under all 100 ROC and PR curves across misclassification levels for each method respectively.

#### Performance of PheLEx when identifying new associations in GWAS

For each simulated misclassified phenotype (in simulations with genetic relatedness/population structure) per misclassification level (1 to 10%), corrected phenotypes were produced using misclassification probabilities estimated by PheLEx. Cases identified as misclassified by PheLEx were switched to controls to result in PheLEx corrected phenotypes. Association analyses were performed for corrected phenotypes produced by PheLEx and resulting *p*-values were used to compute performance metrics. Unadjusted *p*-values computed using association analysis over 100 simulated datasets for each simulated true phenotype (no misclassification), simulated misclassified phenotype, and PheLEx corrected phenotype (at each misclassification level) were used to calculate performance metrics (i.e. recall/true positive rate, false positive rate, and precision). For increasing threshold values (range specified as minimum and maximum unadjusted *p*-values for a given analysis), SNPs with unadjusted *p*-values less than threshold were marked as disease-associated SNPs by the method. Recall/true positive rate was calculated as the fraction of correctly identified disease-associated SNPs out of all disease-associated SNPs in the dataset and false positive rate was calculated as fraction of non-disease associated SNPs with unadjusted *p*-value less than threshold over the total non-disease associated SNPs. Precision was calculated as the fraction of correctly identified disease-associated SNPs in the set of SNPs with unadjusted *p*-values less than threshold. For visualization of ROC curves mean true positive rate across all simulations at each false positive rate value was calculated for association analysis results from simulated true phenotypes (no misclassification), simulated misclassified phenotypes, and PheLEx corrected phenotypes. Similarly, for PR curves, mean precision across all simulations at each recall value was calculated. Area under curve for ROC and PR curves were computed by calculating the area under all 100 ROC curves and 100 PR curves across misclassification levels respectively.

### GWAS case studies: PheLEx analysis of UK Biobank data

#### Case study datasets

Phenotype and genotype data for bipolar disorder and epilepsy were obtained from UK Biobank [141]. Data preprocessing steps used were similar to those adapted in previous analysis for UK Biobank datasets where any differences made no significant impact on results obtained from previous analyses of these GWAS data [89]. For a previous analysis of UK Biobank phenotypes, filtration steps included removing genotypes based on genotyping missingness rate > 2% across samples, MAF < 10^− 4^, and departure from Hardy-Weinberg equilibrium *p* < 10^− 50^, while samples were removed based on missingness rate > 5% across variants, inconsistency between self-reported gender and genetic sex inferred, and non-British white ancestry. For the current analysis of both phenotypes, the UK Biobank dataset was filtered using steps described above with the exception of MAF threshold which was replaced with a more conservative threshold of 10^− 3^. Additionally, all genotypes in LD were pruned from the dataset using PLINK [23] with specific parameters (flag: --indep-pairwise, window size: 50 kb, step size: 5, r^2^ threshold: 0.20). This resulted in 287,425 SNPs in the UK Biobank dataset, which was then divided into datasets for bipolar disorder and epilepsy phenotypes based on diagnosis record. Cases were selected based on diagnosis records where any individual containing diagnosis code for phenotype was labeled a case (bipolar disorder code: 1291 and epilepsy code: 1264). For bipolar disorder, 1177 cases were identified and 3531 controls (three times the number of identified cases) were randomly selected from a pool of individuals who did not have bipolar disorder as their diagnosis in the UK Biobank dataset. For epilepsy, 3620 cases were identified and 10,860 controls (three times the number of identified cases) were randomly selected from a pool of individuals who did not have epilepsy as their diagnosis in the UK Biobank dataset. Distribution of phenotypes and sex within these datasets is described in Table 4. Although previous studies used all the controls provided in the UK Biobank dataset (number of controls = 500,000 – number of cases), number of controls were selected to keep the dataset size manageable and consistent with typical GWAS.

**Table 4:** Distribution of attributes for UK Biobank bipolar disorder and epilepsy datasets

Phenotype	Case	Control	Female	Male
Original bipolar disorder phenotype	1177	3531	2521	2187
PheLEx corrected bipolar disorder phenotype^a^	1123	3585	2521	2187
Original epilepsy phenotype	3620	10,860	7757	6723
PheLEx corrected epilepsy phenotype^a^	3225	11,255	7757	6723

^a^ corrected refers to the dataset where phenotype of individuals identified by PheLEx as misclassified was changed from cases to controls

#### Genome-wide association analysis

The following procedure was used to perform association analyses for simulated phenotypes and real case studies’ phenotypes (UK Biobank bipolar disorder and epilepsy phenotypes). Standard association analysis was applied using a linear mixed model as implemented in R package “lrgpr” [142], where for simulations no additional covariates were used in association analyses whereas for bipolar disorder and epilepsy datasets, GWAS was performed using sex (Male/Female) and age as additional fixed covariates in the association model. The function used for association analyses was lrgprApply, which uses cross-validation and model selection criteria to estimate a genetic relatedness matrix to be used in association analyses [142]. Association analyses for bipolar disorder and epilepsy phenotypes were similar to those performed previously for UK Biobank phenotypes [89], however, some covariates (e.g. batch) previously included [89] were removed from our analyses based on little improvement to quality of Quantile-Quantile plots and GWAS results (Additional file 1: Figure S5). Resulting *p*-values for SNPs from association analyses were also used to filter input for misclassification analyses of simulations and real case studies (Additional file 2).

#### Misclassification analysis of UK Biobank

PheLEx was applied to bipolar disorder and epilepsy datasets to identify potentially misclassified samples in the phenotypes. Consistent with previous GWAS results [89] for both real phenotypes, association analyses failed to produce any statistically significant SNPs using either criteria: (i) Unadjusted *p*-values less than Bonferroni-corrected genome-wide *p*-value threshold or (ii) adjusted *p*-values < 0.1 criteria (*p*-values adjusted using Benjamini-Hochberg procedure). A threshold (unadjusted *p*-value < 10^− 5^) was selected as a heuristic to filter training SNPs used as input for PheLEx for both original bipolar disorder and epilepsy phenotypes (Additional file 1: Text S5). A genetic relatedness matrix was also computed for each dataset using R function getKinship on all SNPs with MAF > 5% [134]. Training SNPs, genetic relatedness matrix, and each original disease phenotype were provided as input to PheLEx. The average misclassification probabilities for cases in each phenotype estimated by PheLEx were used to produce corrected phenotypes for bipolar disorder and epilepsy datasets. PheLEx analysis was performed ten times on each bipolar disorder and epilepsy dataset to produce ten sets of average misclassification probabilities for each phenotype. For bipolar disorder and epilepsy, all samples with average misclassification probability greater than *t* = 95th percentile of misclassification probabilities across sets of misclassification probabilities were marked as misclassified. For both bipolar disorder and epilepsy datasets, phenotypes for samples identified as misclassified were switched from cases to controls to compute corrected phenotypes. Association analyses were performed with corrected phenotypes where SNPs differentially significant according to adjusted *p*-values less than 0.1 (*p*-values were adjusted using Benjamini-Hochberg procedure) and not in LD (r^2^ < *k*, *k* ~ 1e^− 2^) with training SNPs in association analysis with corrected phenotype versus original disease phenotype were considered PheLEx discoveries and investigated for biological significance as described below. Additional details on identification of “misclassified” samples are described in Additional file 1: Text S5.

#### Application of PheLEx to identify new associations

For bipolar disorder and epilepsy datasets, PheLEx was applied on these datasets to estimate misclassification probabilities and compute corrected phenotypes. *P*-values produced from association analyses of corrected phenotypes were adjusted using the Benjamini-Hochberg procedure. SNPs that passed statistical significance threshold (Benjamini-Hochberg adjusted *p*-value < 0.1) were identified and the r^2^ measure of LD was computed between these SNPs and those used for training PheLEx (training SNPs). All non-training SNPs where r^2^ <*k*, *k* ~ 1e^− 2^ with training SNPs and adjusted *p*-value < 0.1 in GWAS with the corrected phenotypes were considered PheLEx discoveries. PheLEx discoveries were further analyzed using web-based resource LDLink [143, 144] where SNPs in LD with PheLEx discoveries were identified and annotated. Additional file 1: Figure S3 was generated using LDLink [143, 144].

## Supplementary information

**Additional file 1.** Supplemental figures, analyses, and algorithm details. We provide details on (i) identifying misclassified samples in datasets simulated without genetic relatedness/population structure, (ii) comparison between PheLEx and other misclassification methods, (iii) effect of differential thresholding on PheLEx’s performance in identifying misclassified samples and GWAS performance, (iv) algorithm details for Rekaya and additional methods, and (v) additional details on UK Biobank phenotypes analyses using PheLEx. We also include supplemental figures referred in the main manuscript.

**Additional file 2. ***P*-values of GWAS for original and PheLEx corrected UK Biobank bipolar disorder and epilepsy phenotypes. We provide *p*-values from GWAS of original bipolar disorder phenotype, PheLEx corrected bipolar disorder phenotype, original epilepsy phenotype, and PheLEx corrected epilepsy phenotype.

## Data Availability

This research has been conducted using the UK Biobank Resource under Application Number 19947. The data underlying the results presented in the study are available from UK Biobank [145]. Results for this study are based on data accessed through the UK Biobank on Feb 21, 2018. The PheLEx software is publicly available as an R package on GitHub [117].

## Abbreviations

AUC PR: Area under precision-recall curve
AUC ROC: Area under receiver operating characteristic curve
AUC: Area under curve
BLMM: Bayesian linear mixed model
GWAS: Genome-wide association studies
LD: Linkage disequilibrium
MAF: Minor allele frequency
MCMC: Markov Chain Monte Carlo
PheLEx: Phenotype latent variable extraction of disease misdiagnosis
PheLEx-mh: PheLEx −/minus Metropolis-Hastings algorithm
PheLEx-mm: PheLEx −/minus mixed model
PR: Precision-Recall
ROC: Receiver operating characteristic
SNP: Single nucleotide polymorphism
