# Supplementary material for: Identifying novel associations in GWAS by hierarchical Bayesian latent variable detection of differentially misclassified phenotypes
Source: BMC Bioinformatics. 2020 May 7;21:178. doi: 10.1186/s12859-020-3387-z (PMC7204256; doi:10.1186/s12859-020-3387-z)
Supplement: Supplementary file 1 — Additional file 1. Supplemental figures, analyses, and algorithm details. We provide details on (i) identifying misclassified samples in datasets simulated without genetic relatedness/population structure, (ii) comparison between PheLEx and other misclassification methods, (iii) effect of differential thresholding on PheLEx’s performance in identifying misclassified samples and GWAS performance, (iv) algorithm details for Rekaya and additional methods, and (v) additional details on UK Biobank phenotypes analyses using PheLEx. We also include supplemental figures referred in the main manuscript. [file 12859_2020_3387_MOESM1_ESM.pdf]

Additional file 1

Text S1. Analysis of datasets simulated without genetic relatedness/population structure

Performance of PheLEx was also investigated in datasets simulated without genetic relatedness or population structure. Genotypes were simulated using "simulateGenotypes" function from R package PhenotypeSimulator[4] for 2,000 samples and 10,000 independent genotypes. Minor allele frequency (MAF) for simulated genotypes was sampled from multinomial distribution with means 0.1, 0.2 and 0.4 (default parameters for "simulateGenotypes" function). One hundred true disease phenotypes (Y') were simulated with 30 randomly selected simulated genotypes using the following relationship:

$$Pr\ (Y' = 1|\beta)\ = \sigma(\beta X + \epsilon)$$
$$\text{where } \epsilon \sim N(0, 1)\ , \beta\ \sim\ N(2, 0.3)$$

Here, σ is probit link function, β are fixed effect sizes of disease-associated SNPs X and ε represents noise. Thirty SNPs were randomly selected to be disease-associated SNPs X for all true phenotypes Y'. Fixed effects β for X were sampled for each disease phenotype separately from normal distribution with mean and variance parameter values stated above. For each simulated true disease phenotype Y', differential misclassification was introduced at varying degrees by switching a fraction of randomly selected controls to cases. Fraction of controls switched to cases varied from 5%, 10%, 20%, 30%, and 40% representing increasing rates of misclassification in "observed phenotype" denoted as Y. Resulting datasets consisted of 100 datasets for each misclassification rate (5%, 10%, 20%, 30%, and 40%).

To investigate benefit of filtering SNPs as input as suggested in the PheLEx framework, the Rekaya[1] method was applied to these simulation datasets using two different inputs: (i) PheLEx input (filtered SNPs using PheLEx's criteria as described in main text) and (ii) unfiltered input where top 1000 SNPs with lowest p-values were used as input. As existing methodologies[1-3] suggest using all SNPs as input for the algorithm, the latter approach provides a way to understand the effect of adding potentially uninformative SNPs as training input. Performances in each case were denoted by Rekaya with PheLEx input and Rekaya respectively. PheLEx-mm was also applied to simulation datasets using built-in filtration criteria. Comparison between Rekaya with PheLEx input and Rekaya (with unfiltered input) provided evidence for improvement in accuracy by filtration of SNPs. PheLEx-mm outperformed both Rekaya with PheLEx input and Rekaya across increasing misclassifications in simulations (Fig. S1). Rekaya (with unfiltered input) performed slightly better than Rekaya with PheLEx input at lower misclassification levels, however, at higher levels of misclassification performance of Rekaya with PheLEx input was superior. It is important to note that this marginal improvement in performance of Rekaya (with unfiltered input) versus Rekaya with PheLEx input is at the expense of computational speed with filtration reducing the time span by 100 times. In contrast, PheLEx-mm was 7 times faster than Rekaya with PheLEx input and 800 times faster than Rekaya (with unfiltered input).

Text S2. Detailed investigation between PheLEx and existing methods

PheLEx-mh, PheLEx-mm, Rekaya (existing method), and PheLEx were applied to simulation datasets to identify misclassified samples from increasingly misclassified simulated phenotypes (%false positives: 1%, 3%, 5%, 8%, and 10%). Differences in performance of PheLEx-mm versus Rekaya and PheLEx versus PheLEx-mh showed improvement due to use of alternative Markov Chain Monte Carlo (MCMC) sampling algorithm (Adaptive Metropolis-Hastings within Gibbs sampling instead of Gibbs Sampling). Comparisons between performance of PheLEx versus PheLEx-mm and PheLEx-mh versus Rekaya showed improvement when accounting for mixed effects in the model. For the simulated datasets with genetic relatedness/population structure across varying degrees of misclassification (1% to 10%), PheLEx outperformed PheLEx-mm, PheLEx-mh, and Rekaya, while PheLEx and PheLEx-mm also showed superior performance to Rekaya and PheLEx-mh (Fig. 2). Area under Receiver Operating Characteristic curve (AUC ROC) and Precision-Recall curve (AUC PR) values in identifying misclassified samples across simulations were also consistent on performance difference observed with PheLEx having the highest median AUC across misclassification levels, where median AUC values (listed in order of increasing misclassification) for PheLEx (median AUC ROC: 0.795, 0.783, 0.773, 0.776, and 0.768; median AUC PR: 0.276, 0.418, 0.471, 0.565, and 0.645), PheLEx-mm (median AUC ROC: 0.58, 0.57, 0.57, 0.56, and 0.56; median AUC PR: 0.038, 0.103, 0.160, 0.227 and 0.264) were higher than median AUC values for PheLEx-mh (median AUC ROC: 0.520, 0522, 0.522, and 0.515; median AUC PR: 0.032, 0.0896, 0.140, 0.203, and 0.239) and Rekaya (median AUC ROC: 0.527, 0.523, 0.522, 0.514, and 0.514; median AUC PR: 0.0323, 0.09, 0.144, 0.205, and 0.240). Use of Adaptive Metropolis-Hastings resulted in the greatest increase in precision over recall as shown by performance of PheLEx and PheLEx-mm versus PheLEx-mh and Rekaya. Though accounting for genetic relatedness/population structure improved performance, this boost was not on the same scale as observed by using alternative MCMC sampling algorithm in realistic simulations.

Using results from applying misclassification extraction methods (i.e., PheLEx, PheLEx-mh, Rekaya and PheLEx-mm) to simulation datasets, we observed running time for each method (Fig. 2). Across all simulations, PheLEx (median time: 0.62 hours) was more efficient than PheLEx-mh (median time: 19 hours) while PheLEx-mm (median time: 0.13 hours) was faster than Rekaya (median time: 6.9 hours). Though accounting for mixed effects adds to running time as shown by comparison between PheLEx-mm and PheLEx, PheLEx's speed was still significantly faster than Rekaya underlining efficiency of our MCMC sampling algorithm of choice. The reason for the increased efficiency of PheLEx compared to Rekaya is that Rekaya implements a full Gibbs Sampler, which requires an update at every iteration for the random effect parameter for every individual and of the effect size parameters of every genotype, along with other updates. In contrast, PheLEx employs an Adaptive Metropolis-Hastings algorithm for sampling effect sizes on genotypes, such that a proposal vector of genotype parameters is simulated and considered for all genotypes simultaneously, instead of a separate update for every genotype. What's more, the random effects in PheLEx sampled through a Gibbs Sampler are only updated when a genotype effect size (vector) parameter proposal is accepted

Text S3. Effect of differential thresholding on performance of PheLEx and Rekaya in simulations

In contrast to earlier assumptions of using all genotypes in the dataset as input to misclassification extraction methods[1-3], filtering of genotypes processed as input (training SNPs) was proposed in this study. The p-value cut-off used to filter training SNPs was $p < 10^{-6.3}$ for performance results stated above and in the results section of the main paper. Once the input was processed by PheLEx and Rekaya respectively, a misclassification probability threshold t was used as cut-off on the average misclassification probabilities (for cases in the phenotype) returned as output. For performance results shown above (and results in the main paper), this threshold t was set to be the 99th percentile of misclassification probabilities, where any sample (case in phenotype) having misclassification probability greater than t was marked as misclassified and switched from case to control in the PheLEx corrected phenotypes produced. Effect of using differential threshold values was examined on (i) the p-value cut-off ($p < 10^{-6.3}$, $p < 10^{-5}$, and $p < 10^{-4}$) used to filter training SNPs, and (ii) the misclassification probability threshold t (99th percentile, 95th percentile, 90th percentile, 85th percentile, 80th percentile, and 75th percentile) used to determine misclassified samples by misclassification extraction methods.

Differential p-value cut-offs for training SNPs impacted the number of training SNPs used as input by misclassification extraction methods across simulations (Fig. S2). Highest number of training SNPs (listed in order of increasing misclassification) was observed for p-value cut-off $p < 10^{-4}$ (median: 26, 25, 22, 21.5, 19, and 19) across misclassification rates, whereas the number of training SNPs decreased as the p-value cut-off became more stringent ($p < 10^{-5}$ median: 9.5, 9, 7, 6, 5.5, and 5; $p < 10^{-6.3}$ median: 4, 3, 3, 2, 2, and 0). Values (listed in order of increasing misclassification) for AUC ROC and AUC PR in identifying misclassified samples by PheLEx ($p < 10^{-6.3}$ AUC ROC median: 0.795, 0.783, 0.773, 0.776, and 0.768; $p < 10^{-6.3}$ AUC PR median: 0.276, 0.418, 0.471, 0.565, and 0.645; $p < 10^{-5}$ AUC ROC median: 0.566, 0.558, 0.550, 0.535, and 0.530; $p < 10^{-5}$ AUC PR median: 0.0504, 0.380, 0.490, 0.646, and 0.663) were superior to those obtained by Rekaya (p $<10^{-6.3}$ AUC ROC median: 0.527, 0.523, 0.522, 0.514, and 0.514; $p < 10^{-6.3}$ AUC PR median: 0.0323, 0.0905, 0.144, 0.205, and 0.240; $p < 10^{-5}$ AUC ROC median: 0.566, 0.558, 0.550, 0.535, and 0.530; $p < 10^{-5}$ AUC PR median: 0.0374, 0.101, 0.153, 0.214, and 0.252) across p-value cut-offs $p < 10^{-5}$ and $p < 10^{-6.3}$.

AUC ROC and AUC PR values improved with increasing misclassification rates across p-value cut-offs, though this improvement was more significant in AUC PR values compared to AUC ROC values. At $p < 10^{-4}$ cut-off for training SNPs, AUC ROC and AUC PR values for Rekaya ($p < 10^{-4}$ AUC ROC median: 0.582, 0.581, 0.562, 0.553, and 0.551; $p < 10^{-4}$ AUC PR median: 0.0408, 0.108, 0.158, 0.224, and 0.264) improved on those for PheLEx ($p < 10^{-4}$ AUC ROC median: 0.475, 0.468, 0.520, 0.490, and 0.474; $p < 10^{-4}$ AUC PR median: 0.0281, 0.0768, 0.143, 0.189, and 0.220), however this improvement was still drastically lower than AUC ROC and AUC PR values for PheLEx at a more stringent p-value cut-off ($p < 10^{-6.3}$). Accompanied by advantages of speed and higher accuracy at more stringent p-value cut-offs, PheLEx showed superior performance in identifying misclassified samples compared to Rekaya at stringent p-value cut-offs.

Impact of misclassification probability threshold t on GWAS performance was also investigated (Fig. S6). Using differential threshold t values (99th percentile, 95th percentile, 90th percentile, 85th percentile, 80th percentile, and 75th percentile) on average misclassification probabilities produced

by PheLEx (with $p < 10^{-6.3}$ p-value cut-off on training SNPs), PheLEx corrected phenotypes were produced at each threshold *t* value across simulations with increasing misclassification rates. Association analyses with the resulting corrected phenotypes at different threshold *t* values were used to compute p-values and used to quantify AUC ROC and AUC PR values in detecting disease-associated SNPs. AUC ROC and AUC PR values in detecting disease-associated SNPs were computed across different threshold *t* values for simulated true phenotypes (no misclassification), misclassified phenotypes, and PheLEx corrected phenotypes for comparison. Across threshold *t* values, with increasing misclassification AUC ROC and AUC PR values in detecting disease-associated SNPs decreased for all phenotypes. At higher misclassification rates, PheLEx corrected phenotypes consistently improved on the AUC ROC and AUC PR values for misclassified phenotypes across threshold *t* values (85th percentile to 99th percentile), however this improvement was not observed at less stringent threshold *t* values (75th percentile and 80th percentile). Gradual decrease in AUC ROC and AUC PR values in detecting disease-associated SNPs with decreasing *t* values can be attributed to the lower precision (Precision = fraction of samples correctly identified as misclassified from total samples identified as misclassified using PheLEx at threshold *t*) in correcting phenotypes at lower values of *t* that result in larger number of true cases switched to controls (Fig. S7), thus lowering the improvement that is observed at more stringent threshold *t* values (that have higher precision of correctly identifying misclassified samples). These results highlight the importance of using stringent threshold on computed misclassification probabilities to determine misclassified samples.

Text S4. Algorithm details for Rekaya, PheLEx-mh, and PheLEx-m

The major existing misclassification framework designed for the analysis of GWAS data is described in three papers [49, 73, 77]. Based on the authorship of these papers, we have designated this method as "Rekaya". Along with comparing PheLEx to the algorithm Rekaya (as described in these publications) we also implemented two additional versions of PheLEx to provide a fair comparison when separately assessing the impact of the two major differences between PheLEx and Rekaya: (i) PheLEx includes an Adaptive Metropolis-Hastings step in the MCMC algorithm not present in Rekaya Gibbs sampler and (ii) PheLEx includes a full mixed model that can account for genetic relatedness/population structure. We have named these two variants of PheLEx as follows:

(i) **PheLEx-mh (PheLEx -/minus Metropolis-Hastings)**: This method removed Adaptive Metropolis-Hastings (mh) step from PheLEx and incorporated the full Gibbs sampler from existing method by Rekaya et al[1] resulting in a full Gibbs sampler with a mixed model

(ii) **PheLEx-mm (PheLEx -/minus mixed model):** This method excluded mixed model from the method PheLEx, resulting in an Adaptive Metropolis-Hastings within Gibbs sampler that doesn't account for mixed effects due to genetic relatedness/population structure.

In the following subsections, we provide details of the implementation of the MCMC algorithms described for the Rekaya framework [49, 73, 77] and the MCMCs for PheLEx-mh and PheLEx-mm.

Rekaya algorithm

The following steps were used from the Bayesian threshold method as described by Rekaya[1]. This method is referred to as Rekaya in simulation results.

1. Initialization
    a. Assume $\alpha = 0$, $\lambda = 0$ with length n_1, n_2 respectively.
    b. Assume $l = y$ *and* $r = y$
    c. Initialize the other parameters with random starting values
2. Update steps
    a. Pr $(\beta_i|\mu, \beta_{-i}, \pi_1, \pi_2, \alpha, \lambda, l_{-i}, r) \sim N(\widehat{\beta_i}, (x_i'x_i)^{-1})$
        i. where $\widehat{\beta_i} = (x_i'x_i)^{-1}x_i'(l_i - 1_n\mu - X_{-i}\beta_{-i})$
    b. $Pr(\mu|\beta, \pi_1, \pi_2, \alpha, \lambda, l, r) \sim N(\sum_{i=1}^{n}(l_i - X_i\beta), \frac{1}{n})$
    c. $Pr(l_i|\mu, \beta, \pi_1, \pi_2, \alpha, \lambda, l_{-i}, r) \sim TN(\widehat{l_i}, 1)$
        i. $\widehat{l_i} = \mu + X_i\beta$
        ii. Distribution is truncated for $l_i > 0$ if $r_i = 1$and $l_i <= 0$ if $r_i = 0$
    d. Estimate misclassified cases
        i. $Pr(\alpha_i = 1|l, \pi_1, y) \propto \frac{\pi_1 p_i(l)^{(1-y_i)}(1-p_i(l))^{y_i}}{K_{\alpha_i}}$
        ii. $Pr(\alpha_i = 0|\beta, \pi_1, y) \propto \frac{(1-\pi_1)(1-p_i(l))^{(1-y_i)}p_i(l)^{y_i}}{K_{\alpha_i}}$
        iii. $K_{\alpha_i} = \pi_1 p_i(l)^{(1-y_i)}(1 - p_i(l))^{y_i} + \pi_1(1 - p_i(l))^{(1-y_i)}p_i(l)^{y_i}$
    e. Estimate misclassified controls
        i. $Pr(\lambda_i = 1|l, \pi_2, y) \propto \frac{\pi_2 p_i(l)^{(1-y_i)}(1-p_i(l))^{y_i}}{K_{\lambda_i}}$
        ii. $Pr(\lambda_i = 0|l_i, \pi_2, y) \propto \frac{(1-\pi_2)(1-p_i(l_i))^{(1-y_i)}p_i(l)^{y_i}}{K_{\lambda_i}}$
        iii. $K_{\lambda_i} = \pi_2 p_i(l)^{(1-y_i)}(1 - p_i(l))^{y_i} + (1 - \pi_2)(1 - p_i(l))^{(1-y_i)}p_i(l)^{y_i}$
    f. Estimate real phenotype
        i. $r_i|y_i, \alpha_i = (1 - \alpha_i)$ (if y_i is case)
        ii. $r_i|y_i, \lambda_i = \lambda_i$ (if y_i is control)
    g. Estimate misclassification probabilities
        i. $\pi_1 = Beta\left(a_1 + \sum_{i=1}^{n_1}\alpha_i, b_1 + n_1 - \sum_{i=1}^{n_1}\alpha_i\right)$
        ii. $\pi_2 = Beta\left(a_2 + \sum_{i=1}^{n_2}\lambda_i, b_2 + n_2 - \sum_{i=1}^{n_2}\lambda_i\right)$
        iii. where hyperparameters are set as $a_1 = a_2 = 1, b_1 = b_2 = 4$
3. Repeat (2) for 99,999 iterations.

Notation

X = genotypes of SNPs

r = real phenotype

y = observed/misclassified phenotype

y_i = observed/misclassified phenotype of individual i

n = total number of samples or individuals

n_1 = total number of cases
n_2 = total number of controls
u = random effects vector representing effect due to genetic relatedness
x_i = column i of X
X_i = row i of X
X_{-i} = matrix X with column i removed
α = vector of misclassification indicator variable for n_1 cases
λ = vector of misclassification indicator variable for n_2 controls
π_1 = misclassification probability in cases
π_2 = misclassification probability in controls
β = SNP effect sizes
β_{-i} = SNP effect sizes vector with element i removed
l_i = liability for individual i
l_{-i} = liability vector with individual i removed
$p_i(l) = p_i(\beta)$ = function of SNP effects
μ = overall mean

PheLEx-mh algorithm

Comparison between PheLEx and PheLEx-mh (PheLEx -/minus Metropolis-Hastings) was used to determine improvement due to use of Adaptive Metropolis-Hastings within Gibbs instead of Gibbs Sampling, and between PheLEx-mh and Rekaya was used to determine improvement when accounting for genetic relatedness. PheLEx-mh was therefore designed to include an underlying mixed model but was implemented without the Adaptive Metropolis-Hastings step within the Gibbs Sampling algorithm, an approach which also provides a fair comparison to existing methods proposed by Rekaya et al[1-3]. Mixed effects due to genetic relatedness/population structure were inferred using conditional probability distributions as described in previous literature[7, 8]. The following steps were used:

1. Initialization
    a. Assume $\alpha = \vec{0}, \lambda = \vec{0}$ with length n_1, n_2 respectively.
    b. Assume $l = y, r = y$
    c. Initialize other parameters with random starting values
2. Update steps
    a. $Pr(\beta_i|\mu, \beta_{-i}, u, \pi_1, \pi_2, \alpha, \lambda, l_{-i}, r, \sigma_u^2) \sim N(\widehat{\beta_i}, (x_i'x_i)^{-1})$
        i. where $\widehat{\beta_i} = (x_i'x_i)^{-1}x_i'(l_i - 1_n\mu - X_{-i}\beta_{-i} - u)$
    b. $\Pr(u_i|\beta, u_{-i}, l, r, \mu, \sigma_u^2, \alpha, \lambda, \pi_1, \pi_2) \sim N(\widehat{u_i}, (1 + c_{ii}\gamma)^{-1})$
        i. $\gamma = ({\sigma_u}^2)^{-1}$
        ii. $\widehat{u_i} = (1 + c_{ii}\gamma)^{-1}\left((l_i - X\beta - \mu) - \gamma c_{i,-i}u_i\right)$
    c. $Pr({\sigma_u}^2|\beta, u, l, r, \mu, \pi_1, \pi_2, \alpha, \lambda, \sigma_u^2) \propto ({\sigma_u}^2)^{-(\frac{q}{2})} exp(-\frac{u'A^{-1}u}{2{\sigma_u}^2}) 1(0, {\sigma_{umax}}^2)$ (7)
    d. $Pr(\mu|\beta, u, \pi_1, \pi_2, \alpha, \lambda, l, r, \sigma_u^2) \sim N(\sum_{i=1}^{n}(l_i - X_i\beta - u_i), \frac{1}{n})$
    e. $Pr(l_i|\mu, \beta, u, \pi_1, \pi_2, \alpha, \lambda, l_{-i}, r, \sigma_u^2) \sim TN(\widehat{l_i}, 1)$
        i. where $\widehat{l_i} = \mu + X_i\beta + u_i$
        ii. Distribution is truncated for $l_i > 0\ if\ r_i = 1$and $l_i <= 0\ if\ r_i = 0$

f. Estimate misclassification in cases using

i. $Pr(\alpha_i = 1|l, \pi_1, y) \propto \frac{\pi_1 p_i(l)^{(1-y_i)}(1-p_i(l))^{y_i}}{K_{\alpha_i}}$

ii. $Pr(\alpha_i = 0|\beta, \pi_1, y) \propto \frac{(1-\pi_1)(1-p_i(l))^{(1-y_i)} p_i(l)^{y_i}}{K_{\alpha_i}}$

iii. where $K_{\alpha_i} = \pi_1 p_i(l)^{(1-y_i)}(1 - p_i(l))^{y_i} + \pi_1(1 - p_i(l))^{(1-y_i)} p_i(l)^{y_i}$

g. Estimate misclassification in controls using:

i. $Pr(\lambda_i = 1|l, \pi_2, y) \propto \frac{\pi_2 p_i(l)^{(1-y_i)}(1-p_i(l))^{y_i}}{K_{\lambda_i}}$

ii. $Pr(\lambda_i = 0|l_i, \pi_2, y) \propto \frac{(1-\pi_2)(1-p_i(l)c)^{(1-y_i)} p_i(l)^{y_i}}{K_{\lambda_i}}$

iii. where $K_{\lambda_i} = \pi_2 p_i(l)^{(1-y_i)}(1 - p_i(l))^{y_i} + (1 - \pi_2)(1 - p_i(l))^{(1-y_i)} p_i(l)^{y_i}$

h. Compute corrected phenotype

i. $r_i|y_i, \alpha = (1 - \alpha_i)$ (if y_i is case)

ii. $r_i|y_i, \lambda = \lambda_i$ (if y_i is control)

i. Update misclassification probabilities

i. $\pi_1 = Beta\left(a_1 + \sum_{i=1}^{n_1} \alpha_i, b_1 + n_1 - \sum_{i=1}^{n_1} \alpha_i\right)$

ii. $\pi_2 = Beta(a_2 + \sum_{i=1}^{n_2} \lambda_i, b_2 + n_2 - \sum_{i=1}^{n_2} \lambda_i)$

iii. where hyperparameters are set as $a_1 = a_2 = 1, b_1 = b_2 = 4$

3. Repeat (2) for 99,999 iterations.

Notation

X = genotypes of SNPs
r = real phenotype
y = observed/misclassified phenotype
A = genetic relatedness matrix
n = total number of samples or individuals
n_1 = total number of cases
n_2 = total number of controls
u = random effects vector representing effect due to genetic relatedness
x_i = column i of X
X_i = row i of X
X_{-i} = matrix X with column i removed
c_{ii} = Element of A^{-1} matrix at row i and column i
$c_{i,-i}$ = row i of A^{-1} with element i removed
u_{-i} = random effects vector u with element i removed
α = vector of indicator variable for n_1 cases
λ = vector of indicator variable for n_2 controls
π_1 = probability of misclassification in cases
π_2 = probability of misclassification in cases
β = SNP effect sizes
β_{-i} = SNP effect sizes vector with element i removed
l_i = liability for individual i
$p_i(l) = p_i(\beta, u)$ = function of SNP effects and random effects at position i
μ = overall mean

σ_u^2 = variance parameter for relatedness

PheLEx-mm Algorithm
This algorithm was designed to exclude an underlying mixed model from PheLEx, where comparison between PheLEx-mm (PheLEx -/minus mixed model) and Rekaya[1-3] was used to determine improvement due to Adaptive Metropolis-Hastings algorithm over Gibbs Sampling without considering effects due to accounting for genetic relatedness/population structure.

Given observed phenotype Y for n individuals and genotypes matrix X for j SNPs

$$Y = [Y_1, \ldots, Y_n]$$
$$X = [\bar{X}_\imath | \ldots | \bar{X}_\jmath]$$

where $\bar{X}_\jmath$ is a genotypes vector for SNP j

For true phenotype (no misclassification) Y' the following is true:

$$Pr(Y'|\beta) \propto \prod_{i=1}^{n} \sigma(\beta)^{Y'_i} \, (1 - \sigma(\beta))^{(1-Y'_i)}$$

where $\sigma(\beta)$ is function of SNP effects

Using this, we can infer the following:

$$Pr(Y|Y', \alpha, \lambda, \beta) \propto Pr(Y|\alpha, \lambda, Y' = 0)\, Pr(Y' = 0|\beta) + Pr(Y|\alpha, \lambda, Y' = 1)\, Pr(Y' = 1|\beta)$$

$$Pr(Y|Y', \alpha, \lambda, \beta) \propto \prod_{i=1}^{n} [\lambda\, (1 - \sigma(\beta)) + \alpha(\sigma(\beta))]^{Y_i} [(1 - \lambda)\, (1 - \sigma(\beta)) + (1 - \alpha)\, (\sigma(\beta))]^{(1-Y_i)}$$

Hence, posterior probability of model is:

$$Pr(Y', \alpha, \lambda, \beta|Y) \propto \prod_{i=1}^{n} [\lambda(1 - \sigma(\beta)) + \alpha\, \sigma(\beta)]^{Y_{i.}} [(1 - \lambda)\big(1 - \sigma(\beta)\big) + (1 - \alpha)\, \sigma(\beta)]^{\,(1-Y_i)}\, Pr(\alpha)\, Pr(\lambda)\, Pr(\beta)$$

To estimate parameters this method employs Adaptive Metropolis-Hastings. This method is referred to as PheLEx-mm in simulations. Parameters (α, λ and β) are estimated in Adaptive Metropolis-Hastings step using the following steps:

1. Initialize random starting values for parameters α, λ and β
2. Define proposal
    a. Sample values for α, λ using truncated normal distribution
    b. Sample values for all β using normal distribution
3. Calculate posterior probabilities from current parameter values and proposal from (2)
    a. Compute $\sigma(\beta)$ for current parameter values and proposal
    b. Compute posterior for current values and proposal using
    $$Pr(Y', \alpha, \beta|Y) \propto \prod_{i=1}^{n} [\lambda(1 - \sigma(\beta)) + \alpha(\sigma(\beta))]^{Y_{i.}} [(1 - \lambda)(1 - \sigma(\beta)) + (1 - \alpha)(\sigma(\beta))]^{(1-Y_i)} Pr(\alpha) Pr(\lambda) Pr(\beta)$$
    c. Update values for parameters with proposal using probability $p = \frac{Posterior\ probability\ with\ proposed\ values}{Posterior\ probability\ with\ current\ values}$
4. Estimate misclassified phenotypes using:
    a. Misclassification in cases ~ Binomial (n_1, p) where

$$p = Pr(Y' = 0|Y = 1, \alpha, \lambda, \beta) = \frac{1}{1+\frac{\alpha\, \sigma(\beta)}{\lambda\, (1-\sigma(\beta))}}$$

b. Misclassification in controls ~ Binomial (n_2, p) where

$$p = Pr(Y' = 1|Y = 0, \alpha, \lambda, \beta) = \frac{1}{1+\frac{(1-\lambda)(1-\sigma(\beta))}{(1-\alpha)(\sigma(\beta))}}$$

5. Repeat from (2) for 99,999 iterations

Variance for jumping distribution of effect sizes is adjusted across iterations to maintain acceptance ratio for MCMC chains around 0.2 using method defined previously[9]. Acceptance rate of 0.2 was used as recommended by Gelman et al[10]. The algorithm was run on each dataset for 100,000 iterations with burn-in of 20,000 iterations. At each iteration, estimates for each parameter are used to calculate misclassification probability for each sample which is then used to identify misclassified samples. Prior probabilities on parameters are defined as:

1. Pr(α) ~ Beta (10, 1)
2. Pr(λ) ~ Beta (1, 1)
3. Pr(β) ~ N (0, 1).

Notation

X = genotypes of SNPs
Y' = real phenotype
Y = observed/misclassified phenotype
n = total number of samples or individuals
n_1 = total number of cases
n_2 = total number of controls
X_i = column i of X
α = probability true positive (observed case is a true case)
λ = probability false positive (observed case is a true control)
β = SNP effect sizes
$\sigma(\beta)$ = function of SNP effects

Text S5. Additional details for analysis of UK Biobank bipolar disorder and epilepsy phenotypes using PheLEx

To ensure reliability of results for UK Biobank phenotypes, a more conservative approach was employed for analyses of these phenotypes using PheLEx. Association analysis was performed for each original disease phenotype and a p-value cut-off used on the resulting unadjusted p-values to filter training SNPs. Using differential p-value cut-offs on training SNPs, number of resulting potential training SNPs were calculated (Fig. S8). As simulation results suggested a conservative threshold, p-value cut-off of 10^{-5} was selected to prune training SNPs used for both bipolar disorder and epilepsy phenotypes. Using training SNPs per phenotype and phenotype data, PheLEx was used to analyze the original disease phenotypes and produce misclassification probabilities. Ten sets of misclassification probabilities were produced by analyzing the data using PheLEx with ten independent chains. All samples identified as misclassified using misclassification probability threshold t (values: 99th percentile, 95th percentile, and 90th percentile) were listed. Any sample marked as misclassified in at least g = 2, 3, 4, 5, and 6 out of ten sets of misclassification

probabilities (produced by PheLEx) were switched from cases to controls for that phenotype. Table S1 lists results from both bipolar disorder and epilepsy phenotypes with threshold *t* values, *g* values (number of misclassification probability sets where sample was consistently marked as misclassified), and the resulting number of cases that were identified as misclassified and switched to controls for that phenotype. Association analysis was performed for each corrected UK Biobank phenotype produced using this methodology and PheLEx discoveries were identified (Table S1). PheLEx discoveries were identified using the same criteria as defined in the main text: PheLEx discoveries were (i) differentially significant SNPs in corrected vs. original UK Biobank phenotype using adjusted p-values < 0.1 as cut-off and (ii) not in LD with training SNPs ($r^2 < k$, $k \sim 1e^{-2}$) used for that phenotype. As simulation results showed increased accuracy with stringent thresholds (*t* and *g*), the most stringent threshold values that resulted in identification of PheLEx discoveries were selected for each phenotype. For PheLEx corrected bipolar disorder phenotype, parameters selected were misclassification probability threshold t = 95th percentile and g = 4 misclassification probability sets. For PheLEx corrected epilepsy phenotype, parameters selected were misclassification probability threshold t = 95th percentile and g = 2 misclassification probability sets. Although additional loci were identified for less stringent threshold *t* and *g* values, a conservative approach was applied and is recommended for future analyses using PheLEx. Results for PheLEx discoveries at selected parameter values are discussed in the main text.

Supplemental Figures

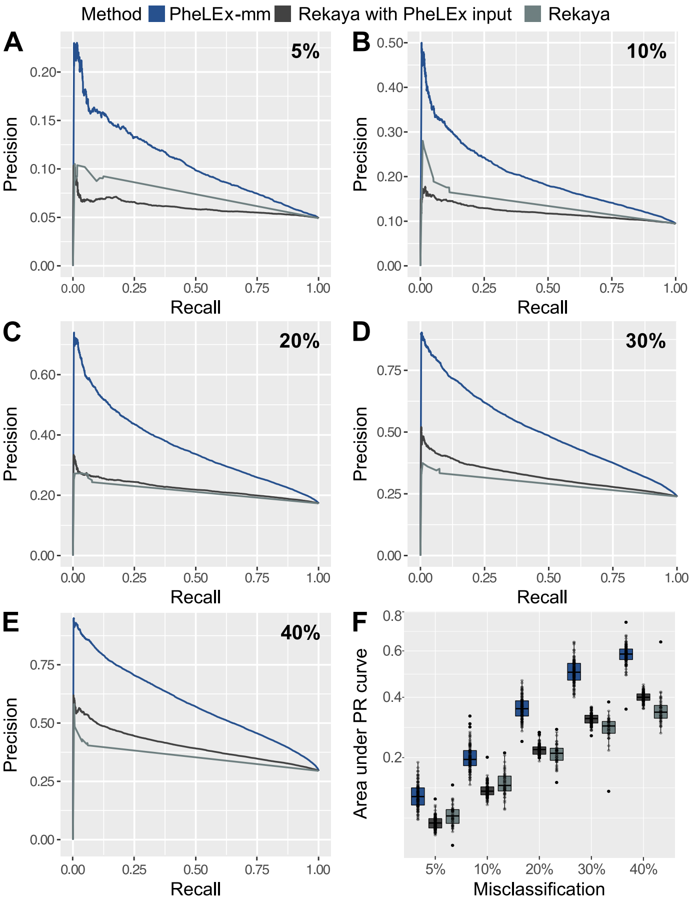

Figure S1. Performance analysis of simulations without genetic relatedness/population structure. (A-E) Mean Precision (y-axis) over Recall (x-axis) over 100 simulated datasets without genetic relatedness/population structure is shown across increasing misclassification (5%, 10%, 20%, 30%, and 40%). Misclassification extraction methods: (i) PheLEx-mm (dark blue) represents PheLEx without mixed model, (ii) Rekaya with PheLEx input (dark gray) represents methodology implemented by Rekaya et al.[1] using filtration of SNPs as performed in PheLEx framework, and (iii) Rekaya (light gray) represents methodology as developed by Rekaya et al.[1] with unfiltered input. (F) Box plots for area under Precision-Recall curves (y-axis) are shown for 100 simulation datasets against increasing misclassification (x-axis) for misclassification extraction methods (Median AUC stated in order of increasing misclassification): PheLEx-mm (dark blue; median AUC: 0.107, 0.195, 0.357, 0.503, and 0.585), Rekaya with PheLEx input (dark gray; median AUC: 0.0609, 0.119, 0.223, 0.322, and 0.400), and Rekaya (light gray; median AUC: 0.0701, 0.129, 0.213, 0.283, and 0.343).

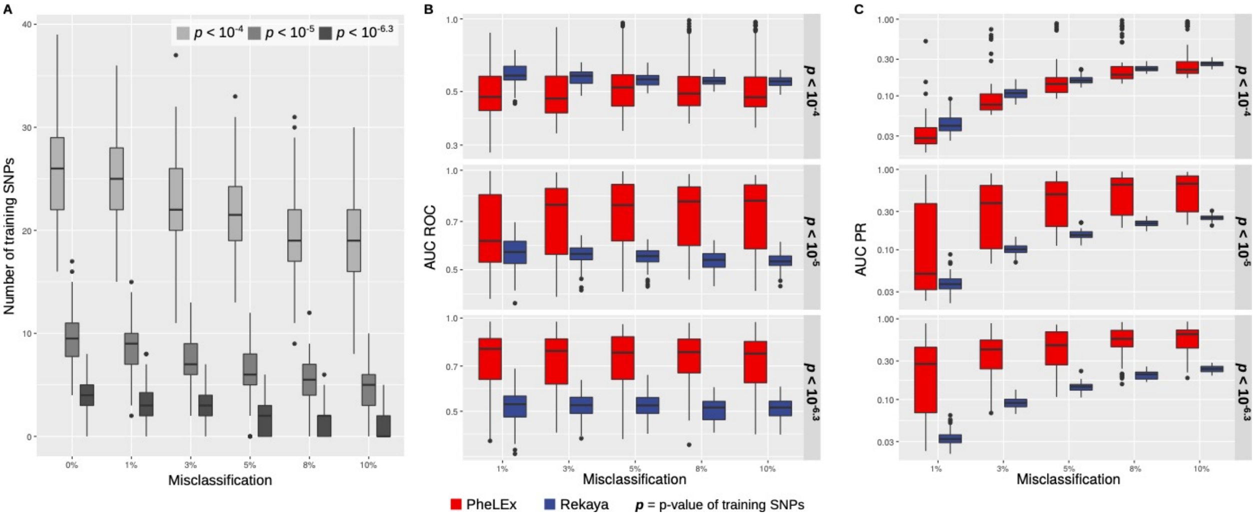

Figure S2. Effect of differential thresholding on training SNPs and performance in simulations. (A) Box plots for number of training SNPs (y-axis) used in misclassification analyses by PheLEx and Rekaya are shown over increasing misclassification rates (x-axis) across different p-value cut-offs used for filtering training SNPs in simulations. (B) Box plots for area under Receiver Operating Characteristic (ROC) curves (AUC ROC) (y-axis) are shown for identifying misclassified samples in simulations against increasing misclassification rates (x-axis) by PheLEx and Rekaya across different p-value cut-offs for filtering training SNPs. (C) Box plots for area under Precision-Recall (PR) curves (AUC PR) (y-axis) are shown for identifying misclassified samples in simulations against increasing misclassification rates (x-axis) by PheLEx and Rekaya across different p-value cut-offs for filtering training SNPs.

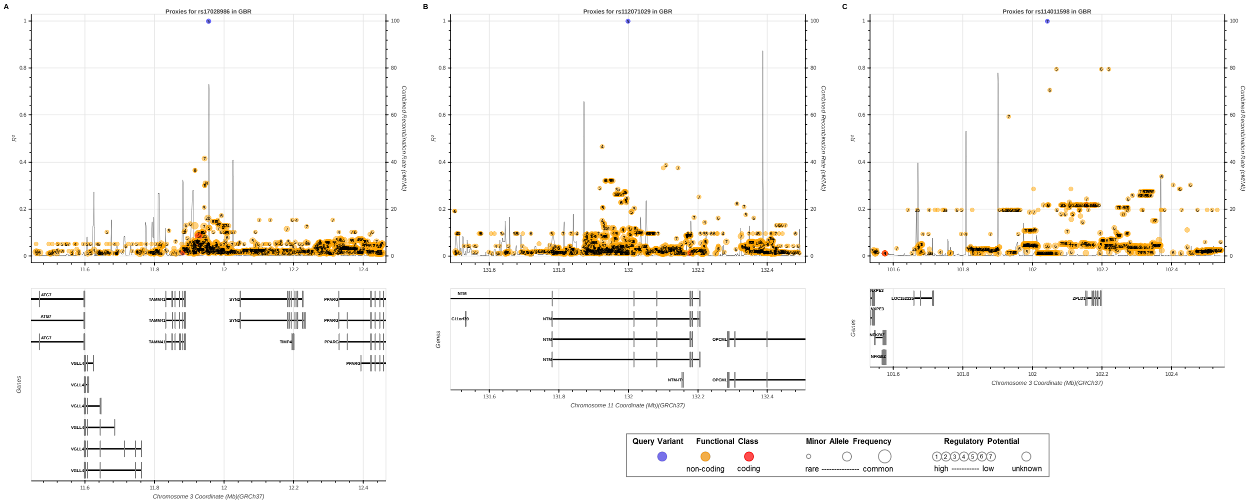

Figure S3. SNPs in linkage disequilibrium with PheLEx discoveries identified for UK Biobank phenotypes. Plots showing linkage disequilibrium (LD) metric r^2 (y-axis on the left), combined recombination rate (y-axis on the right) and chromosome coordinates (x-axis) for SNPs in LD with PheLEx discoveries identified for UK Biobank bipolar disorder phenotype: (A) rs17028986, (B) rs112071029, and epilepsy phenotype: (C) rs114011598.

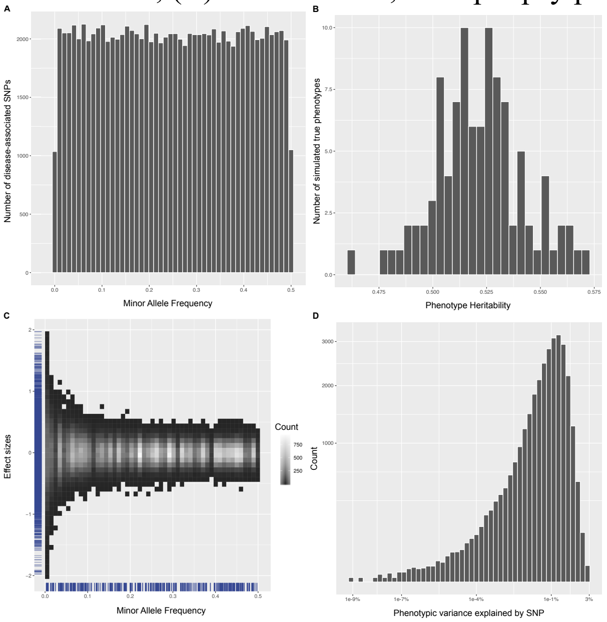

Figure S4. Dataset details for simulation datasets with genetic relatedness/population structure. (A) Histogram showing number of disease-associated SNPs (y-axis) with Minor Allele Frequencies (MAF) (x-axis) across simulations. (B) Histogram showing number of simulated true phenotypes (no misclassification) (y-axis) with total phenotype heritability (x-axis) across simulations. (C) Density plot of simulated effect sizes (y-axis) are shown against MAF (x-axis) for disease-associated SNPs across simulations. Density indicates number of disease-associated SNPs with specified MAF and effect size. (D) Histogram of phenotypic variance explained per SNP (x-axis) is shown against number of disease-associated SNPs (y-axis) across simulations.

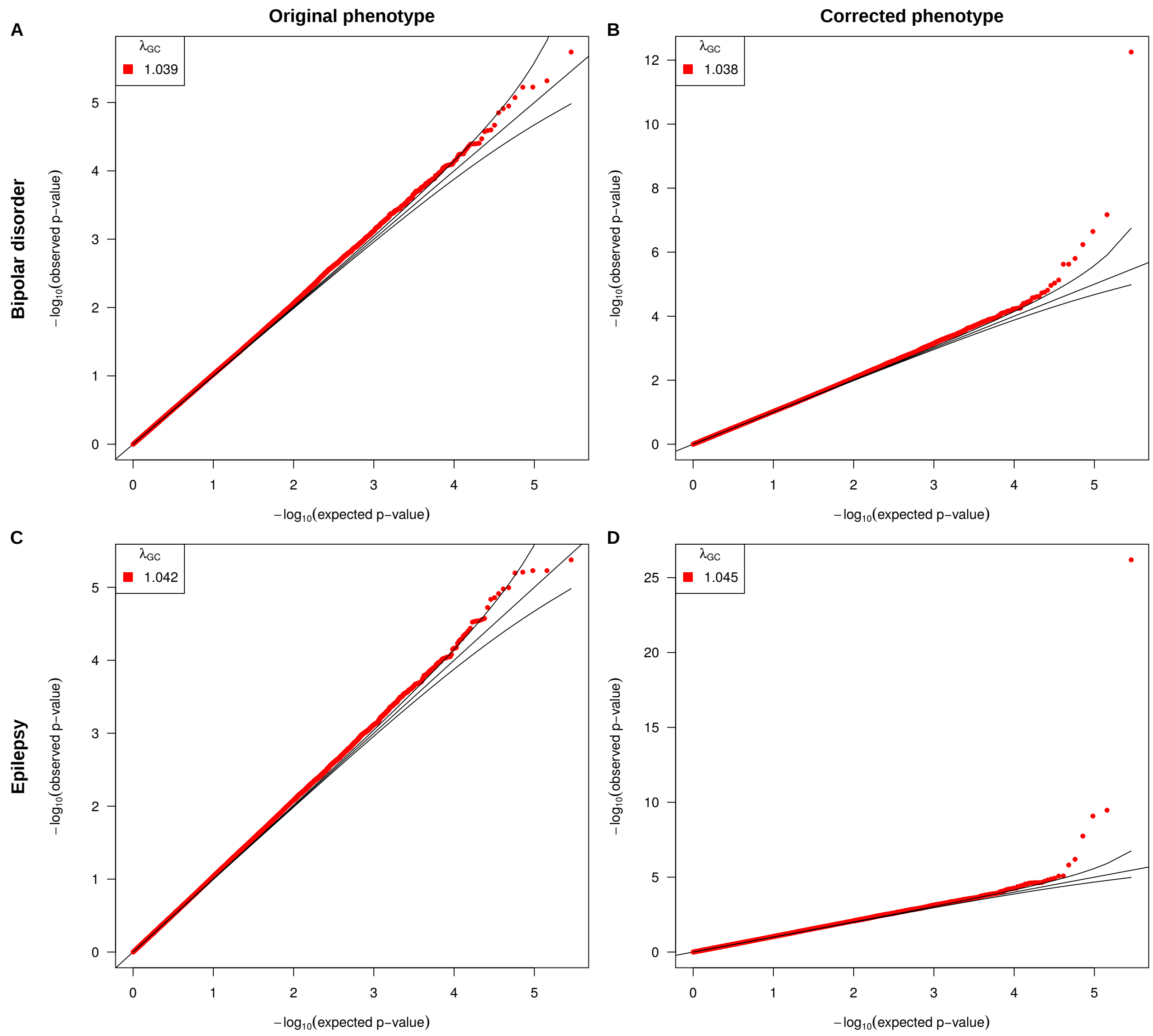

Figure S5. Quantile-Quantile plots for p-values from GWAS of bipolar disorder and epilepsy phenotypes. Quantile-Quantile plots (observed -$\log_{10}$ p-values (y-axis) versus expected -$\log_{10}$ p-values (x-axis)) for GWAS of (A) original bipolar disorder phenotype, (B) PheLEx corrected bipolar disorder phenotype, (C) original epilepsy phenotype, and (D) PheLEx corrected epilepsy phenotype.

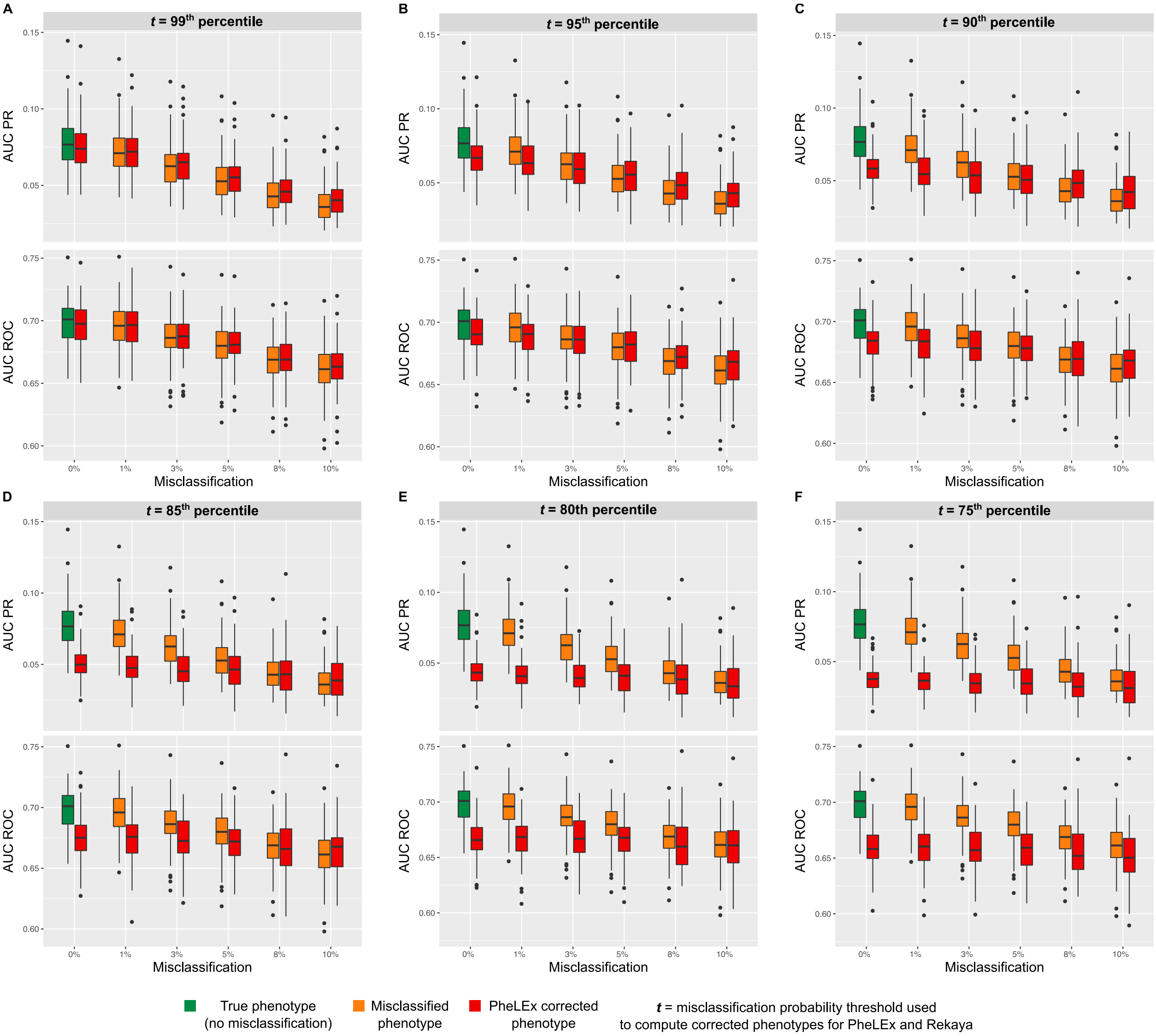

Figure S6. Effect of differential thresholding on GWAS performance in simulations. Box plots for area under Receiver Operating Characteristic (ROC) curves (AUC ROC) (y-axis; bottom) and area under Precision-Recall (PR) curves (AUC PR) (y-axis; top) for detecting disease-associated SNPs using unadjusted p-values obtained from association analyses with simulated true phenotypes (no misclassification) (green), misclassified phenotypes (yellow), and PheLEx corrected phenotypes (red) are shown against increasing misclassification rates (x-axis) across misclassification probability threshold values: (A) $t = 99^{th}$ percentile, (B) $t = 95^{th}$ percentile, (C) $t = 90^{th}$ percentile, (D) $t = 85^{th}$ percentile, (E) $t = 80^{th}$ percentile, and (F) $t = 75^{th}$ percentile.

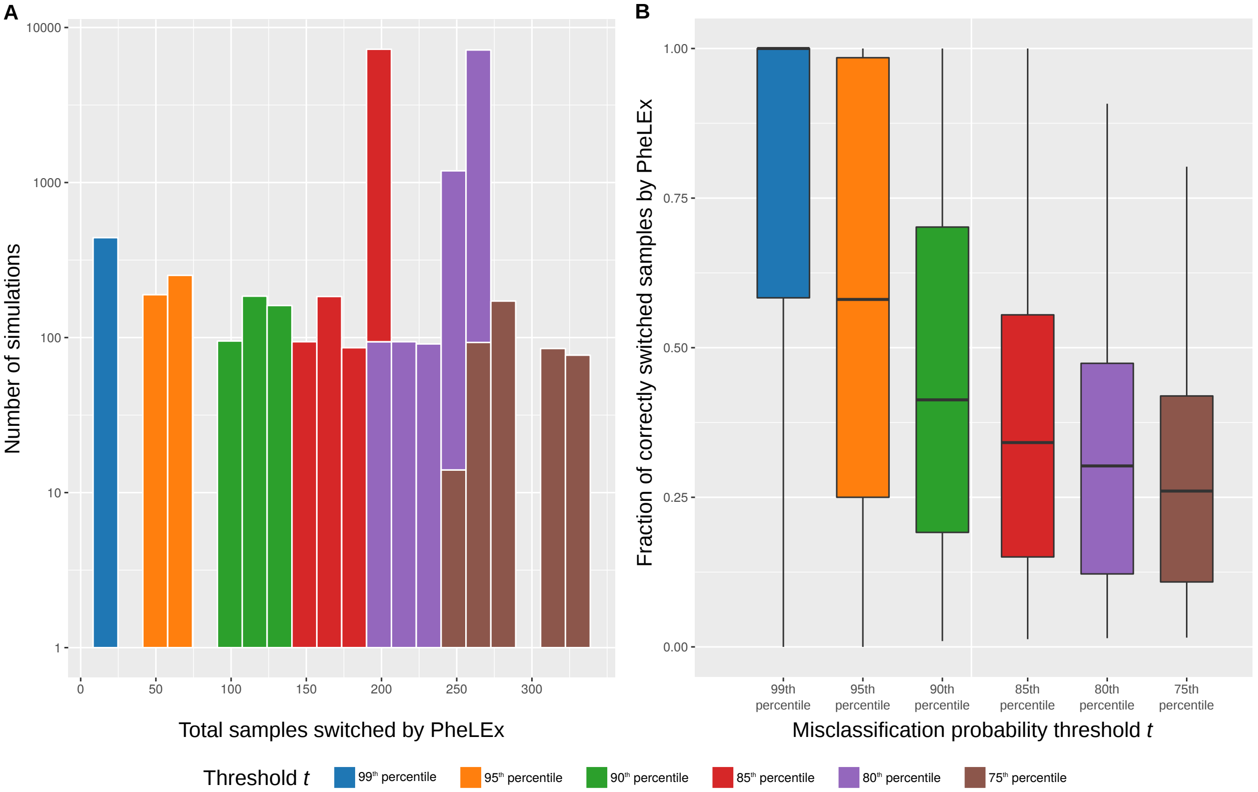

Figure S7. Effect of differential misclassification probability thresholding on samples identified as misclassified in corrected phenotypes across simulations. (A) Histogram of number of simulations (y-axis) against total number of samples identified as misclassified (x-axis) in PheLEx corrected phenotypes is shown across varying values of misclassification probability threshold *t* in simulations. (B) Box plots of fraction of samples correctly identified as misclassified from total samples identified as misclassified (y-axis) in PheLEx corrected phenotypes is shown across varying values of misclassification probability threshold *t* (x-axis) in simulations.

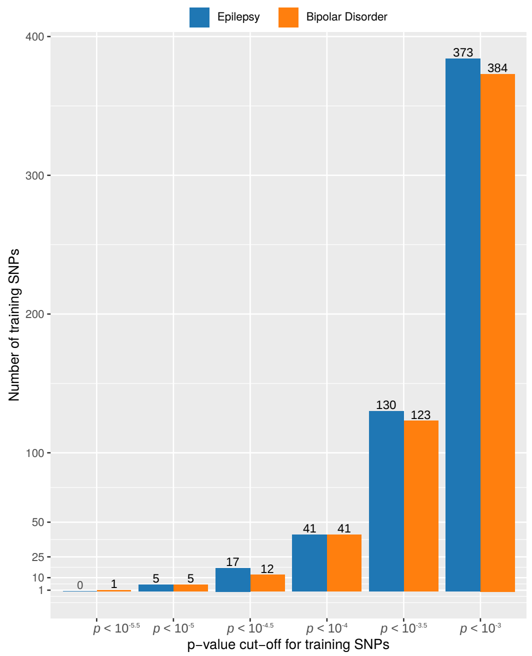

Figure S8. Training SNPs filtered for UK Biobank phenotypes as input for PheLEx. Bar plots showing number of training SNPs (y-axis) as a result of filtering input SNPs (training SNPs) for PheLEx using varying values of p-value cut-off (x-axis) across UK Biobank phenotypes: bipolar disorder (orange) and epilepsy (blue).

Table S1. Effect of differential thresholding of misclassification probabilities on analyses of PheLEx corrected UK Biobank phenotypes

Phenotype	Misclassification probability threshold *t*	No. of PheLEx runs g^a	No. of misclassified samples	No. of differentially significant loci	PheLEx discoveries
Bipolar disorder	99th percentile	2	17	0	
		3	12		
		4	11		
		5	10		
		6	10		
	95th percentile	2	113	1	rs112071029
		3	74	0	
		4	54	2	rs117028986, rs112071029
		5	39	0	
		6	31		
	90th percentile	2	225	2	rs440841, rs4000284,
		3	157	2	rs17028986, rs4000284
		4	117	1	rs4000284
		5	84	1	rs1120701029
		6	64	0	
Epilepsy	99th percentile	2	37	0	
		3	11		
		4	6		
		5	6		
		6	6		
	95th percentile	2	395	1	rs114011598
		3	163	0	
		4	50		
		5	17		
		6	8		
	90th percentile	2	922	2	rs76371186, rs17652437, rs1461822, rs6549871
		3	463	0	
		4	218		
		5	83		
		6	33		

[a] *g* = number of sets of misclassification probabilities produced by PheLEx where the samples were consistently identified as misclassified.

References

1. Rekaya R, Smith S, Hay EH, Farhat N, Aggrey SE: **Analysis of binary responses with outcome-specific misclassification probability in genome-wide association studies**. *Appl Clin Genet* 2016, **9**:169-177.
2. Smith S, Hay el H, Farhat N, Rekaya R: **Genome wide association studies in presence of misclassified binary responses**. *BMC Genet* 2013, **14**:124.
3. Rekaya R, Smith S, Hay el H, Aggrey SE: **Misclassification in binary responses and effect on genome-wide association studies**. *Poult Sci* 2013, **92**(9):2535-2540.
4. Meyer HV, Birney E: **PhenotypeSimulator: A comprehensive framework for simulating multi-trait, multi-locus genotype to phenotype relationships**. *Bioinformatics* 2018, **34**(17):2951-2956.
5. Hoffman GE, Mezey JG, Schadt EE: **lrgpr: interactive linear mixed model analysis of genome-wide association studies with composite hypothesis testing and regression diagnostics in R**. *Bioinformatics* 2014, **30**(21):3134-3135.
6. Runcie DE, Crawford L: **Fast and flexible linear mixed models for genome-wide genetics**. *PLoS Genet* 2019, **15**(2):e1007978.
7. Sorensen DA, Andersen, S., Gianola, D., and Korsgaard, I.: **Bayesian inference in threshold models using Gibbs sampling**. *Genetics, Selection, Evolution* 1995, **17**:229-249.
8. CS Wang JR, and D Gianola: **Bayesian analysis of mixed linear models via Gibbs sampling with an application to litter size in Iberian pigs**. *Genetics Selection Evolution* 1994, **26**(2):91-115.
9. Brooks S. GA, Jones G., and Meng X.-L.: **MCMC Handbook**; 2010.
10. Gelman A. GWR, and Roberts G. O.: **Weak convergence and optimal scaling of random walk Metropolis algorithms**. *Ann Appl Probab* 1997, **7**(1):110-120.
